# Modeling Midbrain and Brainstem Neuromelanins to Characterize Metal Binding and Associated MRI Contrast in Parkinson's and Alzheimer's Diseases

**DOI:** 10.1002/anie.202509102

**Published:** 2025-09-19

**Authors:** Niklas Wallstein, Andrea Capucciati, Andreas Pöppl, Claudia S. Schnohr, Michela Sturini, André Pampel, Carsten Jäger, Luigi Zecca, Fabio A. Zucca, Enrico Monzani, Luigi Casella, Harald E. Möller

**Affiliations:** ^1^ NMR Methods & Development Group Max Planck Institute for Human Cognitive and Brain Sciences Stephanstraße 1A 04103 Leipzig Germany; ^2^ Department of Chemistry University of Pavia Via Taramelli 12 Pavia 27100 Italy; ^3^ Fondazione Pezzoli per la Malattia di Parkinson Via Gianfranco Zuretti 35 Milan 20125 Italy; ^4^ Felix Bloch Institute for Solid State Physics Leipzig University Linnéstraße 5 04103 Leipzig Germany; ^5^ Department of Neurophysics Max Planck Institute for Human Cognitive and Brain Sciences Stephanstraße 1A 04103 Leipzig Germany; ^6^ Paul Flechsig Institute – Centre of Neuropathology and Brain Research Leipzig University Liebigstraße 19 04103 Leipzig Germany; ^7^ Institute of Biomedical Technologies National Research Council of Italy Via Cervi 93, Segrate Milan 20054 Italy

**Keywords:** Bioinorganic chemistry, Copper, Iron, Magnetic resonance, Neuromelanin

## Abstract

Neuromelanin (NM) is a dark pigment that binds potentially toxic metal ions and is crucial for neuronal vulnerability. Magnetic resonance imaging (MRI) was proposed to measure neuromelanin in the *substantia nigra* or *locus coeruleus*, potentially providing a marker of Parkinson's disease. Here, synthetic neuromelanin analogues were prepared with iron and copper and used for characterization of metal binding and impact on proton relaxation, a prerequisite for optimizing neuromelanin‐sensitive MRI. The results confirm the presence of paramagnetic mononuclear Fe(III) and antiferromagnetically coupled clusters, which enhance relaxation to variable degrees. Further complexity arises from Cu(II), which can compete for binding to mononuclear sites, aggregate in mixed‐metal clusters, or bind to proteins associated with the melanin moiety. Unlike the strong relaxant Fe(III), Cu(II) only indirectly impacts relaxation by replacing iron. Overall, MRI primarily provides measures of average neuromelanin concentrations. Information on the distribution of neuromelanins with different metal compositions might be obtained with multiparametric MRI.

## Graphical abstract

Synthetic neuromelanin analogs are used to characterize iron and copper binding to the melanin moiety or to conjugated protein (β‐lactoglobulin). The models confirm the presence of paramagnetic mononuclear‐binding sites and iron or mixed‐metal clusters that enhance water proton relaxation to varying degrees. The results help to better understand MRI contrast changes related to altered neuromelanin content in the brain due to neurodegeneration.

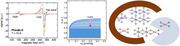

## Introduction

Neuromelanin (NM) is a dark, insoluble pigment found in the central nervous system, particularly in dopaminergic neurons of the *substantia nigra* and noradrenergic neurons of the *locus coeruleus*.^[^ ^1^ ^]^ In both areas, the concentration increases roughly linearly with age.^[^ ^2^, ^3^ ^]^ Neuromelanin is an effective metal‐ion chelator, especially for iron, copper, and zinc.^[^ ^1^, ^3^, ^4^ ^]^ Owing to the ability to bind large amounts of potentially toxic metal ions, a protective role is attributed to NM. Iron is the most abundant metal with up to 11 µg iron/mg NM isolated from *substantia nigra*, whereas NM from *locus coeruleus* binds—in comparison—less iron but more copper.^[^ ^3^, ^5^, ^6^ ^]^ Neuromelanin is also discussed as a critical factor for neuronal vulnerability if saturated with iron or released by degenerating neurons, as in Parkinson's disease (PD) or Alzheimer's disease (AD).^[^ ^4^, ^7^, ^8^, ^9^, ^10^ ^]^ Post‐mortem studies yielded NM levels in PD below 50% compared to healthy controls of the same age.^[^ ^2^ ^]^

Magnetic resonance imaging (MRI) allows an assessment of tissue composition in vivo.^[^ ^11^ ^]^ In the past decade, so‐called neuromelanin‐sensitive MRI (NM‐MRI) has attracted increasing interest as a biomarker in neurodegenerative diseases.^[^ ^12^, ^13^, ^14^, ^15^, ^16^ ^]^ Neuromelanin accelerates the relaxation of water protons due to their paramagnetic properties.^[^ ^17^, ^18^ ^]^ This relaxation enhancement is probably the main source of *T*_1_‐weighted contrast in NM‐MRI, which can be further enhanced by magnetization‐transfer effects in the vicinity of NM‐rich regions.^[^ ^19^, ^20^ ^]^ Alternatively, the effective transverse relaxation rate ($R_{2}^{*}$) and MRI‐based magnetometry were shown to correlate with iron accumulated inside dopaminergic neurons and may provide early markers for PD.^[^ ^21^, ^22^ ^]^

Neuromelanin imaging is challenging due to the small sizes of the *substantia nigra* (∼5 mm thick)^[^ ^23^ ^]^ and *locus coeruleus* (2–3 mm diameter).^[^ ^24^ ^]^ Besides metals, human NM pigment has a melanic, lipid, and protein component.^[^ ^4^, ^25^, ^26^ ^]^ They are all enclosed in specific intraneuronal organelles,^[^ ^1^, ^27^, ^28^ ^]^ making studies of composition and structure difficult. The melanin component consists of insoluble, dark‐brown to black eumelanins and alkali‐soluble, yellow to reddish‐brown pheomelanins with a higher sulfur content.^[^ ^4^, ^29^, ^30^ ^]^ Pheomelanin (Pheo) is thought to form an inner core surrounded by eumelanin (Eu) with an Eu/Pheo ratio of up to ∼3:1.^[^ ^25^, ^31^, ^32^, ^33^ ^]^ Proteins are covalently bound and contribute ∼10% of the pigment mass. They have been partially characterized, showing a fibrillar amyloid cross‐β structure.^[^ ^1^, ^25^ ^]^ The melanin component has a stable free radical that interacts with metals.^[^ ^34^ ^]^ Several studies highlighted two distinct iron‐binding sites in human NM with different affinities.^[^ ^35^, ^36^, ^37^ ^]^ A common feature is that Fe(III) is chelated by catechol oxygen donors in the melanin moiety. Based on electron paramagnetic resonance (EPR) results,^[^ ^3^, ^38^, ^39^ ^]^ rhombic high‐spin Fe(III) centers were assigned to mononuclear sites with low to moderate affinity for the metal, possibly bound to additional water or hydroxo groups.^[^ ^35^, ^39^, ^40^ ^]^ Most iron is likely bound with high affinity through catechol groups in multinuclear clusters, coupled by oxo and/or hydroxo bridges.^[^ ^8^, ^35^, ^38^ ^]^

The interaction of NM and water is of utmost importance for optimizing NM‐MRI acquisitions and understanding the results. This requires information on the impact of binding sites and metal types on relaxation, which is not easily obtained in vivo. Studies of human NM are difficult because only small amounts can be extracted from brains. Synthetic analogues, which can be prepared and modified under well‐defined conditions, help to close this gap. However, mimicking human NM is challenging due to its complexity^[^ ^4^ ^]^ and unknown aspects of the structure. Only a few MRI studies used synthetic melanins, focusing on magnetization transfer.^[^ ^41^, ^42^ ^]^

Compared with earlier studies,^[^ ^43^ ^]^ the current work examined a more realistic model of human NM that contained, besides melanin, β‐lactoglobulin (βLG) as the β‐structured protein component and iron plus copper.^[^ ^3^ ^]^ Little is known about the modulation of MRI parameters by the metal‐ion distribution. Multiple modalities were therefore combined, including inductively coupled plasma optical emission spectrometry (ICP‐OES), X‐ray absorption spectroscopy (XAS), NMR, EPR, and MRI‐based relaxometry and susceptometry. The overarching goal was to better understand how metal accumulation and/or loss of NM associated with neurodegenerative diseases might impact MRI contrast, to aid the development of sensitive methods for detecting disease before symptoms appear.

## Results and Discussion

**Figure 1:**
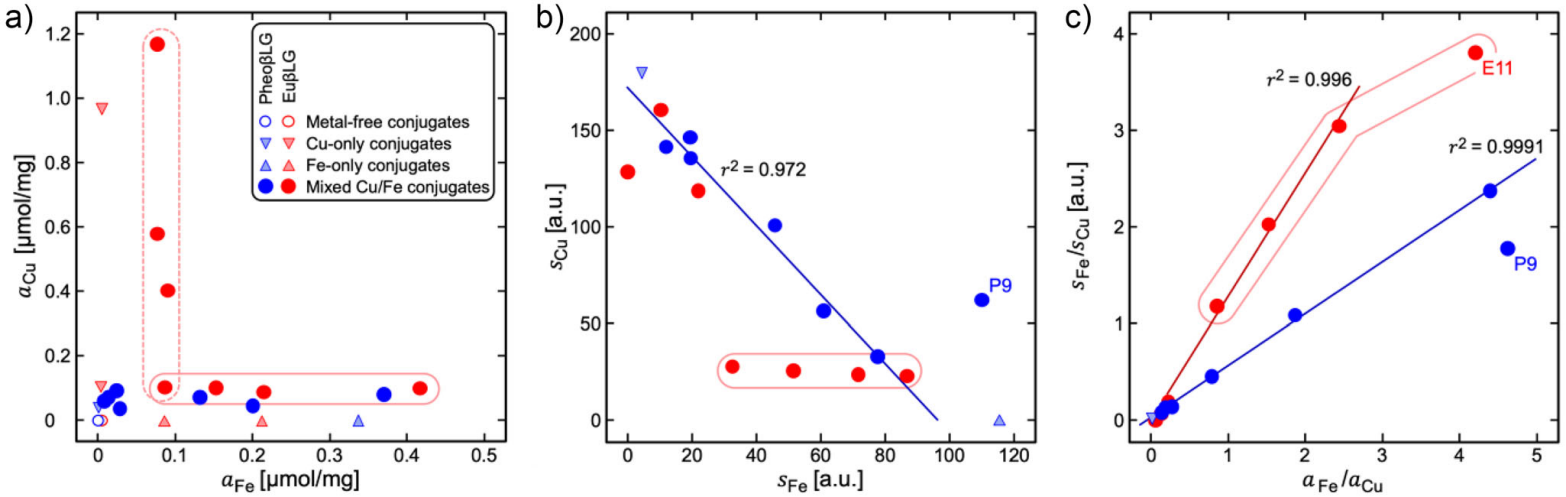
Characterization of the metal content by ICP‐OES and EPR in melanin–protein conjugates. Blue symbols indicate PheoβLG and red symbols EuβLG samples. a) Total Cu content *a*_Cu_ plotted versus total Fe content *a*_Fe_ (both in µmol g^−1^). Mixed‐metal pheomelanins contained only small amounts of Cu (0.036 µmol mg^−1^ ≤ *a*_Cu_ ≤ 0.091 µmol mg^−1^), whereas *a*_Fe_ varied over a large range (0.091 µmol mg^−1^ ≤ *a*_Fe_ ≤ 0.371 µmol mg^−1^). The eumelanin powders were classified as samples with large *a*_Fe_ variation at low *a*_Cu_ (0.088 µmol mg^−1^ ≤ *a*_Cu_ ≤ 0.102 and 0.088 µmol mg^−1^ ≤ *a*_Fe_ ≤ 0.417 µmol mg^−1^; enclosed by solid line) and samples with large *a*_Cu_ variation at low *a*_Fe_ (0.102 µmol mg^−1^ ≤ *a*_Cu_ ≤ 1.168 and 0.077 µmol mg^−1^ ≤ *a*_Fe_ ≤ 0.091 µmol mg^−1^; enclosed by dashed line). b) Variation of the Cu(II) EPR signal area *s*_Cu_ at *g*_e_ = 2.15 with the Fe(III) signal amplitude *s*_Fe_ at *g*_e_ = 4.3 (both measured at 10 K and in arbitrary units, a.u.). Linear regression yielded *s*_Cu_ = (172.4 ± 7.0) − (1.79 ± 0.15)*s*_Fe_ in mixed‐metal pheomelanins (solid blue line). Consistent results in eumelanins were obtained only for high Cu content, whereas samples of low Cu content (enclosed by solid line) showed a distinct deviation from the regression line. c) Fe/Cu ratio, *s*_Fe_/*s*_Cu_, of the EPR‐active metal plotted as a function of *a*_Fe_/*a*_Cu_. A fit to a straight line through the origin yielded slopes of φ = 0.5475 ± 0.0063 and φ = 1.275 ± 0.032 (in a.u.) for PheoβLG‐CuFe (solid blue line) and EuβLG‐CuFe (solid red line), respectively. The samples with the highest iron content, P9 (PheoβLG‐CuFe‐1/10) in b) as well as P9 and E11 (EuβLG‐CuFe‐1/10) in c) were considered outliers and not included in the correlation analyses.

Table 1 lists all samples examined in the current work. They are differentiated by Pheo (for pheomelanin) or Eu (for eumelanin) labels depending on the presence or absence of cysteine, besides dopamine (DA), in the synthesis of the melanic component. Figure 1 provides a graphical representation of the metal contents. The samples were examined as solutions with NMR, as powders with EPR and XAS, or as suspensions in polyacrylamide gel with MRI.

**Table 1:** List of melanin–protein conjugates.

			Metal analysis result (ICP‐OES)					EPR signals	
No.	Sample	% Protein (w/w)	Cu/Fe ratio (w/w)	${\tilde{a}}_{\mathrm{Cu}}\;$(µg mg^−1^)	${\tilde{a}}_{\mathrm{Fe}}\;$(µg mg^−1^)	*a*_Cu_ (µmol g^−1^)	*a*_Fe_ (µmol g^−1^)	*s*_Cu_ (a.u.)	*a*_Fe_ (a.u.)
P0	PheoβLG	56.4 ± 6.5	–	<0.05	<0.05	<0.8	<0.9	0.9	1.9
P1	PheoβLG‐Cu‐5%	57.6 ± 6.2	–	2.5	<0.05	39	<0.9	180.0	4.3
P2	PheoβLG‐Fe‐5%	51.3 ± 4.7	–	<0.05	18.8	<0.8	337	–	115.2
P3	PheoβLG‐CuFe‐1/1	46.0 ± 5.6	1.4/1	2.3	1.6	36	29	100.6	45.7
P4	PheoβLG‐CuFe‐3/1	48.5 ± 3.7	4.1/1	5.8	1.4	91	25	135.6	19.7
P5	PheoβLG‐CuFe‐5/1	60.0 ± 4.0	5.8/1	4.6	0.8	72	14	146.3	19.5
P6	PheoβLG‐CuFe‐10/1	59.3 ± 4.0	7.8/1	3.9	0.5	61	9.0	141.5	12.0
P7	PheoβLG‐CuFe‐1/3	52.5 ± 2.8	1/1.6	4.5	7.4	71	133	56.2	60.9
P8	PheoβLG‐CuFe‐1/5	50.0 ± 7.0	1/3.9	2.9	11.2	46	201	32.7	77.7
P9	PheoβLG‐CuFe‐1/10	40.6 ± 4.5	1/4.0	5.1	20.7	80	371	62.0	110.0
E0	EuβLG	60.0 ± 5.5	–	0.05	0.3	0.8	5.4	–	–
E1	EuβLG‐Cu‐5%	52.0 ± 5.5	–	6.7	0.2	105	3.6	–	–
E2	EuβLG‐Cu‐10%	40.1 ± 6.1	–	61.5	0.3	968	5.4	–	–
E3	EuβLG‐Fe‐5%	56.0 ± 3.5	–	0.02	4.8	0.3	86	–	–
E4	EuβLG‐Fe‐10%	51.6 ± 3.0	–	0.01	11.8	0.2	211	–	–
E5	EuβLG‐CuFe‐1/1	54.2 ± 4.3	1.3/1	6.5	4.9	102	88	27.6	32.5
E6	EuβLG‐CuFe‐3/1	57.6 ± 3.3	5.0/1	25.6	5.1	403	91	118.6	22.0
E7	EuβLG‐CuFe‐5/1	59.0 ± 5.6	8.5/1	36.7	4.3	578	77	160.5	10.4
E8	EuβLG‐CuFe‐10/1	47.6 ± 3.1	17.3/1	74.2	4.3	1168	77	128.3	<0.1
E9	EuβLG‐CuFe‐1/3	55.3 ± 5.0	1/1.3	6.4	8.6	101	154	25.4	51.5
E10	EuβLG‐CuFe‐1/5	58.4 ± 4.2	1/2.1	5.6	12.0	88	215	23.5	71.6
E11	EuβLG‐CuFe‐1/10	51.3 ± 6.5	1/3.7	6.3	23.3	99	417	22.8	86.7

Notations PheoβLG and EuβLG refer to the melanic component obtained from dopamine (DA) in the presence (Pheo) or absence (Eu) of l‐cysteine, respectively, and the protein (βLG) to which melanin is conjugated (1:2 w/w ratio of DA:βLG in the reaction medium). They are followed by the metal type M ∈ {Cu, Fe} and percentage amount (relative to DA) or the Cu/Fe ratio in the reaction medium. These amounts/ratios differ from the concentrations *ã*_M_ in µg mg^−1^ or *a*_M_ in µmol g^−1^ obtained by ICP‐OES in the final samples (relative error ≤ 10%). Further included are the protein fractions in the isolated samples and the *area s*_Cu_ of the Cu(II) resonance at *g* = 2.15 and the *amplitude s*_Fe_ of the Fe(III) resonance at *g* = 4.3 (both measured at 10 K and normalized by the sample mass). Conjugate concentrations in the gel were higher than typical NM concentrations in *substantia nigra* and *locus coeruleus*.^[^ ^2^, ^3^ ^]^ For comparison, Table 2 presents literature values for human NM.

### Characterization of the Conjugate Composition

**Figure 2:**
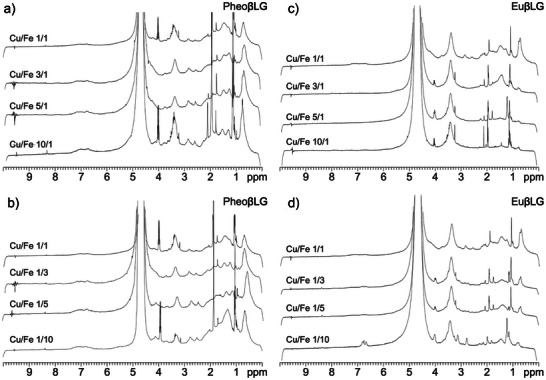
^1^H NMR spectra of synthetic melanins in D_2_O. Spectra of PheoβLG a and b) and EuβLG conjugates c and d) are arranged according to an increasing relative amount of copper (a and c) or iron (b and d) in the reaction medium. They should be compared to those in Figure S4 of Ferrari et al.,^[^ ^46^ ^]^ which also refer to dopamine‐containing melanin‐βLG derivatives.

**Figure 3:**
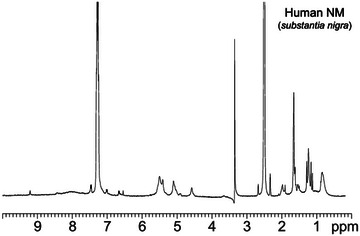
^1^H NMR spectrum of human NM (isolated from *substantia nigra* tissue) in DMSO‐*d*_6_.  ^1^H NMR spectra of human NM isolated from *substantia nigra* in the presence or absence of proteinase K were also published by Double et al.^[^ ^47^ ^]^

NMR spectra of melanin‐βLG conjugates are shown in Figure 2. The aromatic region (6.0–7.5 ppm) contains an almost featureless envelope comprising signals of the melanic component and protein residues. A partial definition of fine structure results with increasing Cu/Fe or Fe/Cu ratio, which may be associated with increased conformational mobility of the melanic component. Changes were also found at 1.0–1.5 ppm (aliphatic side chains of βLG residues), showing a broad signal that splits into two components with increasing Cu/Fe ratio. Spectra of samples with excess iron over copper do not show a recognizable trend and need further scrutiny. An NMR spectrum of human NM isolated from *substantia nigra* tissue is shown in Figure 3 for comparison.

The melanic moiety is covalently bound to βLG, preventing proteolysis at the expected sites. Therefore, missing peptide fragments in liquid chromatography‐mass spectrometry (LC‐MS) of synthetic conjugates resulting from proteolysis probably contain the binding sites for DA quinones and are covalently linked to melanic oligomers. In the present conjugates (Table S1), His146 seems to be the βLG primary binding site of DA‐quinones, as Cys66 and Cys106 are involved in disulfide bridges,^[^ ^44^, ^46^ ^]^ whereas Cys121 is not accessible in the native protein.

### EPR Measurements

**Figure 4:**
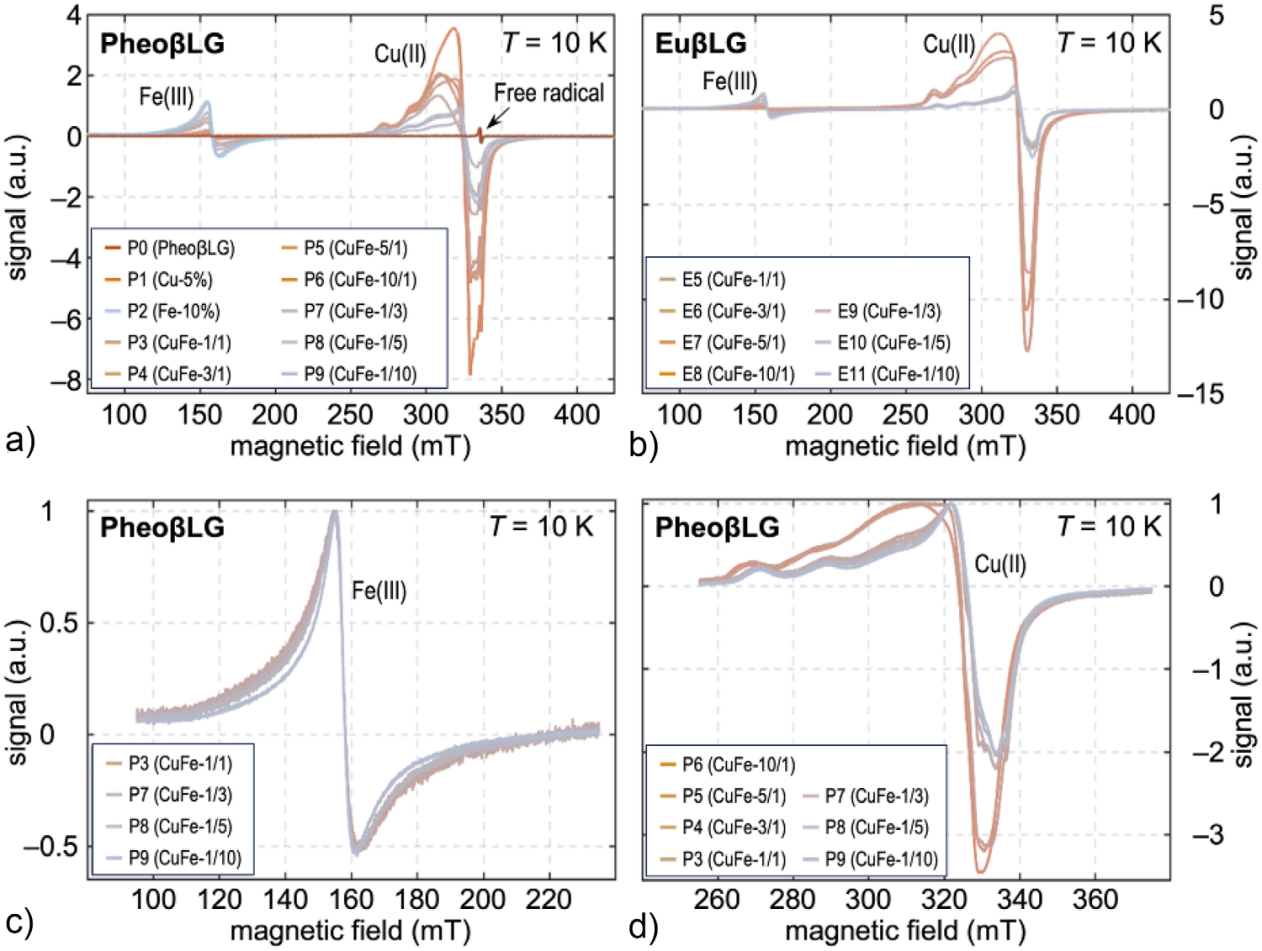
Characterization of melanin–protein conjugates by EPR recorded at 10 K. The variation of the metal content is indicated by the line color. Scaled X‐band EPR spectra of ten PheoβLG‐CuFe a) and seven EuβLG‐CuFe powders b) are shown as well as lineshape details of the Fe(III) resonance at *g* = 4.3 c) and the Cu(II) resonance at *g* = 2.15 d) in exemplarily selected PheoβLG samples. Note that the spectra in a) and b) have been scaled according to the receiver gain and the sample mass to ensure comparability of the recording, whereas the amplitudes of the signals in (c and d) were normalized to 1 for better visibility of the lineshape differences. Main acquisition parameters are summarized in Table S2.

Three main features are evident in EPR spectra recorded at 10 K (Figure 4): i) A sharp peak of the free radical (*g*‐factor 2.0 in metal‐free samples) that is partially masked by a strong Cu(II) resonance at *g* ≈ 2.15; ii) hyperfine splitting of the Cu(II) signal; iii) a high‐spin Fe(III) signal with rhombic symmetry and large zero‐field splitting at *g* = 4.3 as in human NM (Table 2).^[^ ^1^, ^38^, ^39^ ^]^ Both signal amplitudes decreased with increasing temperature (60 K and room temperature). This would be expected for Curie–Weiss paramagnetism, which was demonstrated for synthetic neuromelanins^[^ ^38^, ^48^ ^]^ and also for Fe(III) and Cu(II) resonances in lyophilized brain tissue.^[^ ^49^ ^]^ However, data from only three temperatures is insufficient to confirm this assumption in comparison to other possible factors (e.g., a change in the *Q*‐factor of the cavity). Room‐temperature measurements (Figure S1) indicated another very broad iron signal at *g* = 2.1 aggregated in an antiferromagnetic domain,^[^ ^38^, ^48^ ^]^ which was undetectable at 10 K. This is similar to results in human ferritin, where a regime of progressive unblocking of magnetic moments with increasing temperature was demonstrated.^[^ ^50^ ^]^

**Table 2:** Properties of human NM pigment isolated from the *substantia nigra* and the *locus coeruleus*, as reported in the literature.^[^ ^3^, ^25^, ^34^, ^39^, ^44^, ^45^ ^]^

		Metal content			EPR *g*‐factors		
Brain region	% Protein (w/w)	Cu/Fe ratio (w/w)	${\tilde{a}}_{\mathrm{Cu}}\;$(µg mg^−1^)	${\tilde{a}}_{\mathrm{Fe}}\;$(µg mg^−1^)	Free radical	Cu(II)	Fe(III)
*Substantia nigra*	8.8 ± 2.3	1/58.9	0.19 ± 0.02	10.9 ± 1.4	2.0	≈2.3	4.3
*Locus coeruleus*	–	1/2.9	0.61 ± 0.08	1.78 ± 0.09	2.0	≈2.3	4.3

Protein content (*n* = 3 samples)^[^ ^25^ ^]^ as well as Cu and Fe contents (*n* = 3 samples)^[^ ^3^, ^39^ ^]^ are given as total aminoacidic content (%) and total metal content in µg/mg dry pigment, respectively (mean ± standard error of the mean, SEM).^[^ ^25^ ^]^ Unlike the synthetic NM preparations, the isolated pigment contained bound lipids,^[^ ^25^ ^]^ which were part of the dry weight to which the concentrations refer, that is, the numerical values are systematically lower compared to Table 1. Further shown are *g*‐factors of paramagnetic EPR signals in human NM pigment without further information on the relative amounts of EPR‐active Cu and Fe, which were not determined in these works.^[^ ^34^, ^44^, ^45^ ^]^

The Fe(III) signal showed minimal lineshape variations at 10 K, which allowed to estimate relative Fe(III) amounts from peak amplitudes. The Cu(II) lineshape was more variable, consistent with reports of composition‐dependent hyperfine parameters, probably indicating mixed oxygen/nitrogen coordination.^[^ ^44^ ^]^ These changes are particularly evident in samples with excess copper, suggesting a distribution among multiple sites and, at least at low copper loading, binding to the protein. Figure 4d demonstrates an apparent shift of the partially resolved parallel hyperfine component *g*_||_ toward higher values with increasing copper load, suggesting that excess Cu(II) binds to sites richer in oxygen donors. Iron(III) displayed a pure oxygen coordination due to its high affinity for oxygen donors.

### EPR‐Active and Total Metal Contents

The EPR‐active amounts *s*_Fe_ of Fe(III) and *s*_Cu_ of Cu(II) were highly correlated in PheoβLG conjugates (Figure 1b; squared Pearson coefficient *r* ^2^ = 0.972). Results in EuβLG‐CuFe were consistent for high copper content, whereas *s*_Cu_ was apparently independent of *s*_Fe_ for low copper content.

Similarly, the ratio *s*_Fe_/*s*_Cu_ demonstrated a strong linear correlation with the ratio of the total metal contents, *a*_Fe_/*a*_Cu_, for PheoβLG (Figure 1c; *r* ^2^ = 0.9991). The same result was obtained for the inverse ratios (Figure S2). Figure 1c indicates similar linearity for EuβLG, albeit with different scaling. However, the double‐reciprocal plot (*s*_Fe_/*s*_Cu_ vs. *a*_Fe_/*a*_Cu_) is relatively insensitive for deviations at high *a*_Cu_, which becomes clearer when plotting *s*_Cu_/*s*_Fe_ against *a*_Cu_/*a*_Fe_ (Figure S2). This difference between both melanins mirrors the observation of different regimes in EuβLG‐Cu/Fe with high and low *a*_Cu_ (Figure 1b).

Features of the relationships between EPR‐active and total metal contents were (Figure S3): i) *s*_Fe_ increased steadily with *a*_Fe_ and leveled off at high iron content; ii) *s*_Cu_ increased with *a*_Cu_ leveling off at high copper load for EuβLG‐CuFe, but varied between different PheoβLG‐CuFe samples despite similar *a*_Cu_; iii) *s*_Fe_ showed no clear dependence on *a*_Cu_ for PheoβLG‐CuFe and tended to lower values at high *a*_Cu_ for EuβLG‐CuFe; iv) *s*_Cu_ decreased with increasing *a*_Fe_, but was independent of *a*_Fe_ for EuβLG‐CuFe with low *a*_Cu_. These characteristics suggest multiple types of mononuclear binding sites in melanin‐βLG conjugates, possibly with competition between Fe(III) and Cu(II) for the same sites.

**Figure 5:**
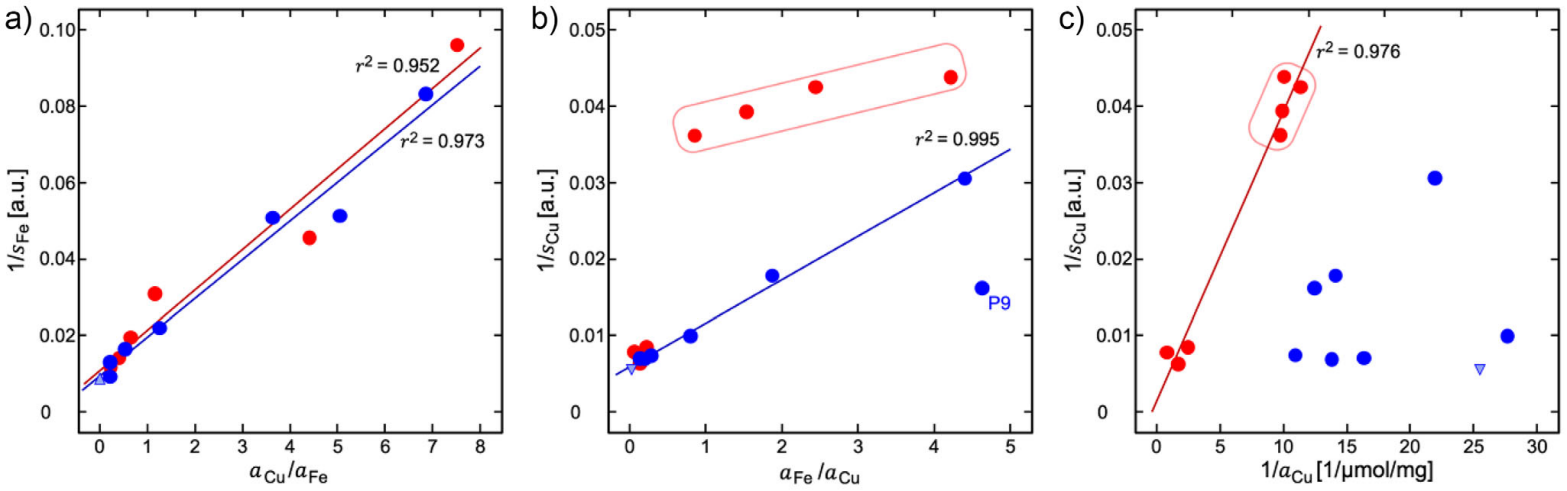
Lineweaver–Burk plots describing the dependencies of the EPR signals measured at 10 K on the metal content. Blue and red symbols indicate pheomelanin and eumelanin conjugates, respectively, as in Figure 1. a) Variation of 1/*s*_Fe_ obtained from the EPR signal of Fe(III) at *g* = 4.3 as a function of *a*_Cu_/*a*_Fe_ and results from fits to Eq. S9 for the limiting case of *c*_0_ *a*_Fe_ + *c*_2_ *a*_Cu_ ≫ *c*_1_ (i.e., conditions of competition and high metal content) shown as solid lines. b) Variation of 1/*s*_Cu_ obtained from the EPR signal of Cu(II) at *g* = 2.15 as a function of *a*_Fe_/*a*_Cu_. A fit to Eq. S11 for the limiting case of *c*_0_ *a*_Fe_ + *c*_2_ *a*_Cu_ ≫ *c*_1_ describes the experimental result for PheoβLG‐CuFe (solid blue line; sample P9 regarded as outlier) but not in EuβLG‐CuFe samples of low Cu content (enclosed by solid red line). c) Variation of 1/*s*_Cu_ as a function of 1/*a*_Cu_. A fit to Eq. S11 for the limiting case of *c*_1_ + *c*_2_ *a*_Cu_ ≫ *c*_0_ *a*_Fe_ (i.e., negligible competition) approximately describes the experimental result for EuβLG‐CuFe (solid line) but not for PheoβLG‐CuFe.

Employing a Langmuir model without competition, *s*_Fe_ was approximately described by assuming saturation at high *a*_Fe_ (albeit less well for EuβLG‐CuFe; Figure S4). It improved (particularly for EuβLG‐CuFe) when binding competition with Cu(II) was considered (Figure 5) according to (cf. Eqs. S7–S9):

(1) $$s_{\mathrm{Fe}}=\frac{a_{\mathrm{Fe}}}{c_{1}+c_{0}a_{\mathrm{Fe}}+c_{2}a_{\mathrm{Cu}}}\approx \frac{a_{\mathrm{Fe}}}{c_{0}a_{\mathrm{Fe}}+c_{2}a_{\mathrm{Cu}}}\;\mathrm{if}\;c_{0}a_{\mathrm{Fe}}+c_{2}a_{\mathrm{Cu}}\gg c_{1}.$$

Fitted parameters (units of [*s*_Fe_]^−1^) were *c*_0_ = 0.0091 ± 0.0023 and *c*_2_ = 0.01019 ± 0.00069 for PheoβLG‐CuFe as well as *c*_0_ = 0.0108 ± 0.0043 and *c*_2_ = 0.0106 ± 0.0012 for EuβLG‐CuFe (Table 3). Competition becomes negligible if *c*_1_ + *c*_0_ *a*_Fe_ ≫ *c*_2_ *a*_Cu_. Restricting the analysis to five samples with lowest *a*_Cu_/*a*_Fe_ yielded a rough estimate of *c*_1_ = 0.0004 ± 0.0001 (units of [*a*_Fe_]/[*s*_Fe_]) under this assumption.

**Table 3:** Langmuir‐model and relaxation parameters (at room temperature). *c*_0_, *c*_0_, and *c*_2_ characterize the binding of iron or copper ions to pheomelanin (Pheo) or eumelanin (Eu); φ is a scaling parameter. *R*_1,0_, Δ *R* and $r_{1,\mathrm{Fe}}^{m}$ are the longitudinal relaxation rate of the metal‐free melanin‐βLG preparation, the longitudinal relaxation enhancement due to metal clusters and the longitudinal relaxivity due to mononuclear Fe(III), respectively. *R*_2,0_, Δ *R*, and $r_{2,\mathrm{Fe}}^{m}$ are the corresponding transverse relaxation parameters.

		Value		
Parameter	Unit	Pheo	Eu	Reference
*c*_0_	[*s*_Fe_]^−1^	0.0091 ± 0.0023	0.0108 ± 0.0043	Eq. 1
*c*_1_	µmol g–1/[*s*_Fe_]	0.0004 ± 0.0001		Eq. 1
*c*_2_	[*s*_Fe_]^−1^	0.01019 ± 0.00069	0.0106 ± 0.0012	Eq. 1
φ*c*_0_	[*s*_Cu_]^−1^	0.00572 ± 0.00017	–	Eqs. 2 and 3
φ*c*_1_	µmol g–1/[*s*_Cu_]	–	0.0013 ± 0.0021	Eq. 3
φ*c*_2_	[*s*_Cu_]^−1^	0.00587 ± 0.00032	0.00379 ± 0.00026	Eq. 2
*R*_1,0_	s^−1^	0.503 ± 0.004	0.661 ± 0.009	Eq. 7
$\Delta R_{1p}^{c}$	s^−1^	0.182 ± 0.028	0.135 ± 0.043	Eq. 7
$r_{1,\mathrm{Fe}}^{m}$	s^−1^/[*s*_Fe_]	(2.01 ± 0.61) × 10^−3^	(4.41 ± 0.85) × 10^−3^	Eq. 7
*R*_2,0_	s^−1^	1.32 ± 0.01	1.71 ± 0.02	Eq. S3
$\Delta R_{2p}^{c}$	s^−1^	0.97 ± 0.07	0.58 ± 0.07	Eq. S3
$r_{2,\mathrm{Fe}}^{m}$	s^−1^/[*s*_Fe_]	(2.7 ± 1.2) × 10^−3^		Eq. S3

An equivalent competition model (cf. Eq. S11),

(2) $$s_{\mathrm{Cu}}\approx \frac{a_{\mathrm{Cu}}}{\varphi \left(c_{0}a_{\mathrm{Fe}}+c_{2}a_{\mathrm{Cu}}\right)},$$

yielded an excellent fit for PheoβLG‐CuFe with φ*c*_0_ = 0.00572 ± 0.00017 and φ*c*_2_ = 0.00587 ± 0.00032 (both in units of [*s*_Cu_]^−1^). Consistent results were obtained for EuβLG‐CuFe with high but not low *a*_Cu_ (Figure 5b). A model without competition,

(3) $$s_{\mathrm{Cu}}\approx \frac{a_{\mathrm{Cu}}}{\varphi \left(c_{1}+c_{2}a_{\mathrm{Cu}}\right)},$$

yielded acceptable fits for EuβLG‐CuFe (but not for PheoβLG‐CuFe; Figure 5c) with φ*c*_1_ = 0.0013 ± 0.0021 and φ*c*_2_ = 0.00379 ± 0.00026 (in units of [*a*_Cu_]/[*s*_Cu_] and [*s*_Cu_]^−1^, respectively), albeit with limited significance due to the distribution of the data in two blobs (high and low *a*_Cu_).

The EPR results demonstrate that only a small portion of the iron in the melanin preparations is paramagnetic. This agrees with studies suggesting EPR‐active Fe(III) fractions below 5% in mixed Cu/Fe derivatives and somewhat higher values in those containing only iron.^[^ ^43^, ^44^, ^46^ ^]^ Most iron is, therefore, aggregated in clusters with antiferromagnetic coupling (i.e., a reduced effective electron spin *S*), resembling human NM. A reduced EPR‐active Cu(II) fraction (9%–52% in mixed Cu/Fe derivatives) was also found previously,^[^ ^44^ ^]^ corroborated by our results.

The correlations in Figure 1 suggest that Fe(III) and Cu(II) can bind to identical mononuclear sites in PheoβLG‐CuFe. The agreement of a competitive‐adsorption model with the dependence of *s*_Fe_ on *a*_Fe_/*a*_Cu_ (Figure 5a) confirms this assumption. Remarkably, the fitted parameters *c*_0_ and *c*_2_ suggest similar binding affinities for both ions.

In EuβLG‐CuFe, there appears to be at least one additional binding site for EPR‐active Cu(II) that is preferentially (and independently of Fe) populated at low *a*_Fe_ (Figure 1b). For higher *a*_Cu_ but relatively low *a*_Fe_, the variation of *s*_Cu_ resembled the behavior in PheoβLG‐CuFe. Although the combined data could be fitted to a noncompetitive model (Figure 5c), this result is of limited significance because a single binding site does not seem to cover the entire Cu‐concentration range in EuβLG‐CuFe. While iron is expected to bind exclusively to the melanic part, copper can also bind to the protein. Due to the higher protein/melanin ratio (∼1:1) in synthetic compared to human NMs (Tables 1 and 2),^[^ ^46^ ^]^ this may be enhanced in our model systems. Unlike Fe(III), Cu(II) does not exhibit a strong tendency to aggregate in medium/large homonuclear clusters. The possibility that Cu populates positions close to Fe (clusters) was recently discussed, particularly, at the interface of the protein and melanic parts, leading to coupled Cu–Fe sites.^[^ ^44^ ^]^ Such coupling might reduce the EPR Cu(II) signal without full cancellation. The decreasing *s*_Cu_ at higher *a*_Fe_ (Figure 5b) could result from increasing formation of heteronuclear instead of homonuclear (iron) clusters. Aggregation in different cluster types would in turn alter the Cu(II) EPR lineshape and probably contributes to the pronounced lineshape differences observed in Figure 4d.

### XAS Measurements

**Figure 6:**
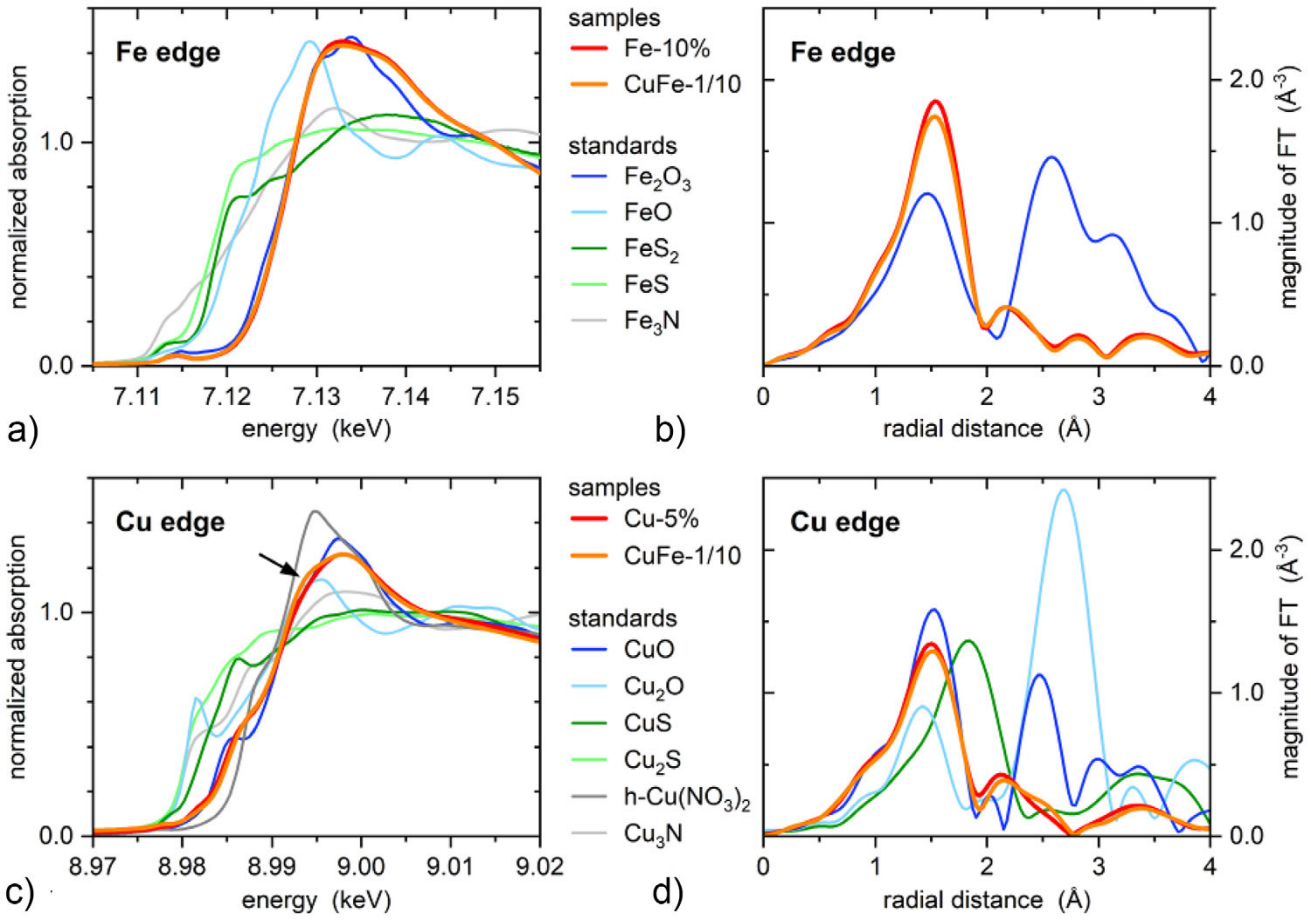
Characterization of melanin‐protein conjugates by XAS at 10 K. a) Fe K‐edge XANES spectra and b) magnitude of *k* ^2^‐weighted Fourier transforms (FT) of the EXAFS for PheoβLG‐Fe‐10%, PheoβLG‐CuFe‐1/10, and Fe_2_O_3_. c) Cu K‐edge XANES spectra and d) magnitude of the *k* ^2^‐weighted FT of the EXAFS for PheoβLG‐Cu‐5%, PheoβLG‐CuFe‐1/10, CuO, Cu_2_O, and CuS. The black arrow indicates a shoulder at ∼8.994 keV. Room‐temperature XANES spectra for FeO, FeS_2_, FeS, and Fe_3_N in a) and for Cu_2_S, Cu(NO_3_)_2_· 3H_2_O [here referred to as h‐Cu(NO_3_)_2_] and Cu_3_N in c) were taken from the MDR XAFS database (see Supporting Information). Different temperatures do not affect the XANES but preclude comparing the EXAFS.

The X‐ray absorption near edge structure (XANES) depends on the electronic structure and local geometry around the absorbing element and is a fingerprint for the oxidation state, coordination number, symmetry, and bond length. Unlike EPR, XAS is sensitive to all atoms of an element, representing an average over all binding sites. Iron K‐edge XANES spectra for PheoβLG‐Fe‐10% and PheoβLG‐CuFe‐1/10 were almost identical (Figure 6a). They resembled the spectrum for Fe_2_O_3_, whereas other standards showed different edge positions and shapes. Accordingly, the best fit by linear combination analysis (LCA) was obtained with 100% Fe_2_O_3_. Remaining differences above the absorption edge (7.13–7.15 keV) probably stem from disorder and structural differences beyond the first coordination shell.^[^ ^51^ ^]^ The samples thus feature Fe(III)‐O local environments without significant amounts of Fe(II)‐O, Fe(II)‐S, or Fe(IV)‐S.

The extended X‐ray absorption fine structure (EXAFS) depends on local structural parameters, including bond length, coordination number, and disorder. The magnitude of the Fourier‐transformed Fe K‐edge EXAFS data for PheoβLG‐Fe‐10%, PheoβLG‐CuFe‐1/10, and Fe_2_O_3_ showed a strong peak at ∼1.5 Å (Figure 6b), which originates from scattering of photoelectrons at oxygen atoms in the first coordination shell. Fe_2_O_3_ exhibits further scattering contributions from higher coordination shells at 2–4 Å. They were strongly dampened or absent for the conjugates, indicating disorder beyond the first coordination shell. Structural parameters were obtained using a path‐fitting approach for the peak at ∼1.5 Å. Fe_2_O_3_ features three short and three long Fe─O bonds with fitted EXAFS bond lengths of 1.94 ± 0.01 and 2.09 ± 0.01 Å at 10 K. For both conjugates, an excellent fit was obtained with six oxygen nearest neighbors and one common bond length of 1.996 ± 0.002 Å (Figure S5), in agreement with previous XAS in human and synthetic neuromelanins.^[^ ^52^ ^]^ The disorder parameter was similar to those in Fe_2_O_3_, indicating a well‐ordered first coordination shell with pseudo‐octahedral symmetry. FeO also features octahedral coordination but with a significantly larger crystallographic distance of 2.162 Å.^[^ ^53^ ^]^ Replacing oxygen by nitrogen in the structural model yielded a similar fit of the peak at ∼1.5 Å. Therefore, the EXAFS analysis cannot distinguish between oxygen and nitrogen nearest neighbors. Adding sulfur, iron, or copper or including oxygen as second coordination shell yielded indeterminate structural parameters regarding their uncertainties. The EXAFS analysis thus confirms Fe(III)‐O local environments without significant amounts of Fe(II)─O or Fe─S bonds.

Copper K‐edge XANES spectra for PheoβLG‐Cu‐5% and PheoβLG‐CuFe‐1/10 were nearly identical, except for a more pronounced shoulder at ∼8.994 keV for PheoβLG‐CuFe‐1/10 (Figure 6c). The edge position and shape resembled those of CuO—albeit with larger discrepancy than for Fe XANES—and were distinctly different from results in other standards. Best LCA fits were obtained with an admixture of up to ∼10% Cu_2_O, CuS, Cu_2_S, or Cu_3_N (Table S3), with remaining discrepancy. Adding Cu(NO_3_)_2_·3H_2_O did not improve the fit for PheoβLG‐Cu‐5% but yielded the best result for PheoβLG‐CuFe‐1/10 with a better representation of the shoulder (Figure S5). PheoβLG‐Cu‐5% thus featured Cu(II)‐O local environments, possibly with small fractions of Cu(II)‐S or Cu(I)‐S, whereas PheoβLG‐CuFe‐1/10 likely consisted of ∼80% Cu(II)‐O and ∼20% Cu(II)‐N local environments.

The scattering contribution due to an oxygen first coordination shell in the Fourier‐transformed Cu K‐edge EXAFS was again observed at 1.4–1.5 Å whereas the signal for Cu─S bonds occurred at ∼1.8 Å, indicating mostly Cu─O bonds (Figure 6d). Contributions from higher coordination shells were strongly dampened, demonstrating disorder beyond the first coordination shell as for iron. CuO features four Cu─O bonds with average fitted EXAFS bond lengths at 10 K of 1.946  ±  0.003 Å. For PheoβLG‐Cu‐5% and PheoβLG‐CuFe‐1/10, an excellent fit was obtained with four oxygen nearest neighbors and Cu─O bond lengths of 1.932  ±  0.003 and 1.944  ±  0.003 Å, respectively (Figure S5). The difference persisted independent of the fit settings, indicating slightly different Cu nearest‐neighbor environments. Both samples had identical disorder parameters, roughly twice as high as for CuO, demonstrating higher disorder already within the first coordination shell. Nitrogen and oxygen nearest neighbors could not be distinguished, whereas adding sulfur, iron, or copper to the local structure did not yield meaningful fits. Including oxygen atoms as second coordination shell fitted the side peak at ∼2.2 Å (Figure S5), however, with high uncertainties of the associated structural parameters and unchanged parameters of the first coordination shell. The EXAFS analysis thus confirms Cu(II)‐O [or Cu(II)‐N] local environments without relevant amounts of Cu─S bonds.

Interestingly, there was no difference between PheoβLG‐Fe‐10% and PheoβLG‐CuFe‐1/10 regarding the local iron environment but slight differences in the Cu XANES and EXAFS between PheoβLG‐Cu‐5% and PheoβLG‐CuFe‐1/10. This could be due to differences in the preferred iron and copper binding sites or related to different iron and copper loadings (Table 1). As both PheoβLG‐Fe‐10% and PheoβLG‐CuFe‐1/10 feature high *a*_Fe_, the small amount of copper in PheoβLG‐CuFe‐1/10 does not affect the average local coordination of iron. In contrast, both PheoβLG‐Cu‐5% and PheoβLG‐CuFe‐1/10 feature low *a*_Cu_, and the high *a*_Fe_ in PheoβLG‐CuFe‐1/10 could impact the copper coordination, especially if both metals compete for the same sites.

### Proton Relaxation and Bulk Magnetic Susceptibility

Water relaxation and the bulk magnetic susceptibility Δχ were examined for associations with *a*_Fe_ and *a*_Cu_, the amount of EPR‐active high‐spin Fe(III) and Cu(II), and the total metal content, expressed as effective magneton concentration that would result for uncoupled “spin‐only” paramagnetic ions:

(4) $$p=\sqrt{35}a_{\mathrm{Fe}}+\sqrt{3}a_{\mathrm{Cu}}.$$

**Figure 7:**
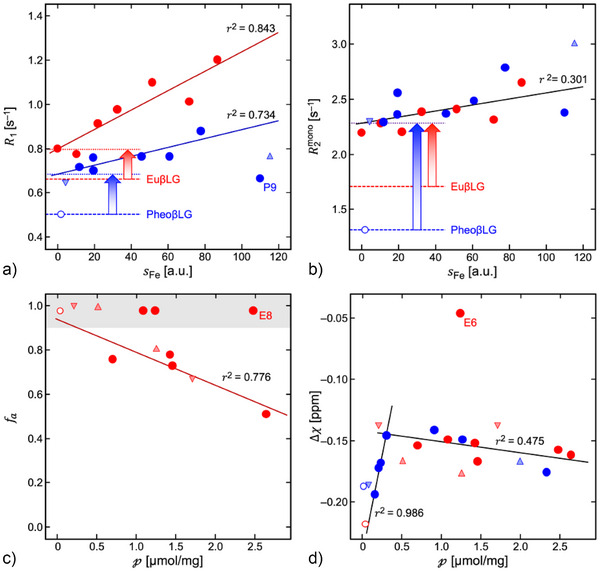
Proton relaxation rates and bulk magnetic susceptibility at room temperature in dependence of the metal content. Blue and red symbols indicate pheomelanin and eumelanin conjugates, respectively, as in Figure 1. Variation of a) *R*_1_ and b) *R* 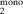 obtained by monoexponential fitting with the amount of EPR‐active Fe(III) (measured at 10 K). Linear regression in mixed‐metal conjugates (solid lines) yielded *R*_1_/s^−1^ = (0.685 ± 0.028) + (0.00201 ± 0.00061)*s*_Fe_ and *R*_1_/s^−1^ = (0.796 ± 0.042) + (0.00441 ± 0.00085)*s*_Fe_ in PheoβLG‐CuFe and EuβLG‐CuFe, respectively, as well as *R*_2_/s^−1^ = (2.285 ± 0.066) + (0.0027 ± 0.0012)*s*_Fe_ for the combined data from both melanins. Blue and red arrows show the *R*_1_ or *R* 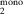 difference between the metal‐free conjugate (see Table S4) and the extrapolation of the regression line toward *s*_Fe_ = 0. c) Negative correlation of the fraction of the slow‐relaxing compartment with the total metal content (here expressed as magneton concentration p). Estimates of *f_a_* in the gray shaded area were considered less reliable and excluded in the analysis. d) Variation of the bulk magnetic susceptibility with the total metal content. The Δχ values reflect the susceptibility difference between the melanin/gel suspensions and the agarose gel doped with (paramagnetic) gadopentetate dimeglumine surrounding the samples (see Figure S6). The resulting negative values, hence, do not mean that the conjugates are diamagnetic.

Longitudinal relaxation times at 3 T are summarized in Figure S6 and Table S4. The presence of protein reduced *T*_1_ of the gel by 3%–5%. Metal‐free eumelanin caused further shortening. Stronger effects resulted with metal‐containing conjugates (*T*_1_ reductions by 20%–41% in PheoβLG and 16%–53% in EuβLG). Paramagnetic relaxation enhancement (PRE) was stronger at 3 T than at 7 T (on average by 58%; Figure S7) and more pronounced for dominating iron content. The longitudinal rate, *R*_1_ = 1/*T*_1_, increased approximately linearly with *s*_Fe_ as expected for classical PRE (Figure 7). Linear regression yielded a relaxivity $r_{1,\mathrm{Fe}}^{m}=(2.01\pm 0.61)\times 10^{-3}$ (units of s^−1^/[*s*_Fe_]) assigned to mononuclear Fe(III) in PheoβLG‐CuFe (Table 3). The relaxation enhancement due to multinuclear clusters was estimated as $\Delta R_{1p}^{c}=\;0.182\pm 0.028$ s^−1^ from the difference between the intercept and *R*_1,0_ = 0.503 ± 0.004 s^−1^ in the metal‐free samples. The same analysis for EuβLG‐CuFe yielded $r_{1,\mathrm{Fe}}^{m}=(4.41\pm 0.85)\times 10^{-3}$ (units of s^−1^/[*s*_Fe_]), $\Delta R_{1p}^{c}\approx 0.135\pm 0.043$ s^−1^ and *R*_1,0_ = 0.661 ± 0.009 s^−1^. Remarkably, *R*_1_ appeared to *decrease* with *increasing* amounts of EPR‐active Cu(II) (Figure S8). This apparent “negative relaxivity” is quantitatively explained by co‐variation of *s*_Fe_ with *s*_Cu_ indicating minimal PRE from mononuclear Cu(II). *R*_1_ changes with *a*_Fe_, *a*_Cu_, or p were also well approximated by concomitant changes in *s*_Fe_. This suggests a largely constant contribution to *R*_1_ from metal clusters.

The transverse rate $R_{2}^{\mathrm{mono}}=1/T_{2}^{\mathrm{mono}}$, estimated with monoexponential analysis also increased with increasing *s*_Fe_ (Figure 7b). Linear regression yielded a relaxivity $r_{2,\mathrm{Fe}}^{m}=(2.7\pm 1.2)\times 10^{-3}$ (units of s^−1^/[*s*_Fe_]) assigned to mononuclear Fe(III) for both melanins and contributions of $\Delta R_{2p}^{c}=0.97\pm 0.07$ s^−1^ and 0.58 ± 0.07 s^−1^ due to clusters estimated from the intercept and *R*_2,0_ = 1.32 ± 0.01 s^−1^ and 1.71 ± 0.02 s^−1^ of metal‐free PheoβLG and EuβLG, respectively (Table 3). As for *R*_1_, apparent $R_{2}^{\mathrm{mono}}$ changes with *s*_Cu_, *a*_Fe_, or *a*_Cu_ were explained by co‐varying *s*_Fe_ (Figure S9). Additional measurements in EuβLG‐CuFe revealed deviations from monoexponential behavior. They were fitted to a biexponential model with fractions *f*_a_ and *f*_b_ = (1 − *f*_a_) and rates $R_{2}^{a}$ and $R_{2}^{b}$ of ‘slow‘ and ‘fast‐relaxing’ water compartments, respectively. $R_{2}^{a}$ agreed roughly with the monoexponential result (Table S4). The corresponding fraction dominated at low metal content (*f*_a_ > 0.9) but decreased with increasing p (Figure 7c). The increasing $R_{2}^{\mathrm{mono}}$ in Figure 7b thus reflects a growing contribution from the faster‐relaxing compartment.

Figure 7d indicates an initial increase of Δχ with increasing metal loading up to a tipping point around p=0.5 µmol mg^−1^, followed by a slow decrease. This probably reflects a dominant superparamagnetic contribution from clusters and a smaller contribution from mononuclear binding sites, which might be of relatively greater importance at low metal content.

### Model for Water Proton Relaxation

Our results replicate reports of only minor effects on water relaxation by metal‐free NM models,^[^ ^41^, ^54^ ^]^ mainly because the free‐radical concentration is too low for a substantial impact.^[^ ^18^ ^]^ The seemingly unsystematic variation of relaxation rates with metal loading in both conjugate types can be described by assuming a largely invariant contribution of Fe clusters—which may also contain coupled Cu—and a linear PRE from mononuclear Fe(III). This latter fraction is assigned to high‐spin paramagnetic centers.^[^ ^28^, ^45^ ^]^ Remarkably, there was no relevant PRE from EPR‐active Cu(II). Due to their electron configurations, a more efficient relaxivity is expected for high‐spin Fe(III) (*S* = 5/2) than for Cu(II) (*S* = 1/2). For an *inner‐sphere mechanism* ^[^ ^55^ ^]^ mediated by transient bonding of water and/or proton exchange between solvent water and coordinating hydroxy groups, this difference between both ions may be further modulated by differences in the electronic spin relaxation times, ion‐proton distances or water‐molecule lifetimes in the inner sphere. Consistently, our experimental data suggest

(5) $$a_{\mathrm{Cu}}^{m}r_{1,\mathrm{Cu}}^{m}\ll a_{\mathrm{Fe}}^{m}r_{1,\mathrm{Fe}}^{m}\;\mathrm{and}\;a_{\mathrm{Cu}}^{m}r_{2,\mathrm{Cu}}^{m}\ll a_{\mathrm{Fe}}^{m}r_{2,\mathrm{Fe}}^{m},$$

where

(6) $$a_{M}^{m}=\kappa_{M}s_{M}$$

is the concentration of ion M∈{Cu,Fe} bound to a mononuclear site and κ_M_ a scaling factor. This allows to approximate *R*_1_ as (cf. Eqs. S1–S7):

(7) $$R_{1}=R_{1,0}+\Delta R_{1p}^{c}+\kappa_{\mathrm{Fe}}r_{1,\mathrm{Fe}}^{m}\frac{a_{\mathrm{Fe}}}{c_{1}+c_{0}a_{\mathrm{Fe}}+c_{2}a_{\mathrm{Cu}}}.$$

**Figure 8:**
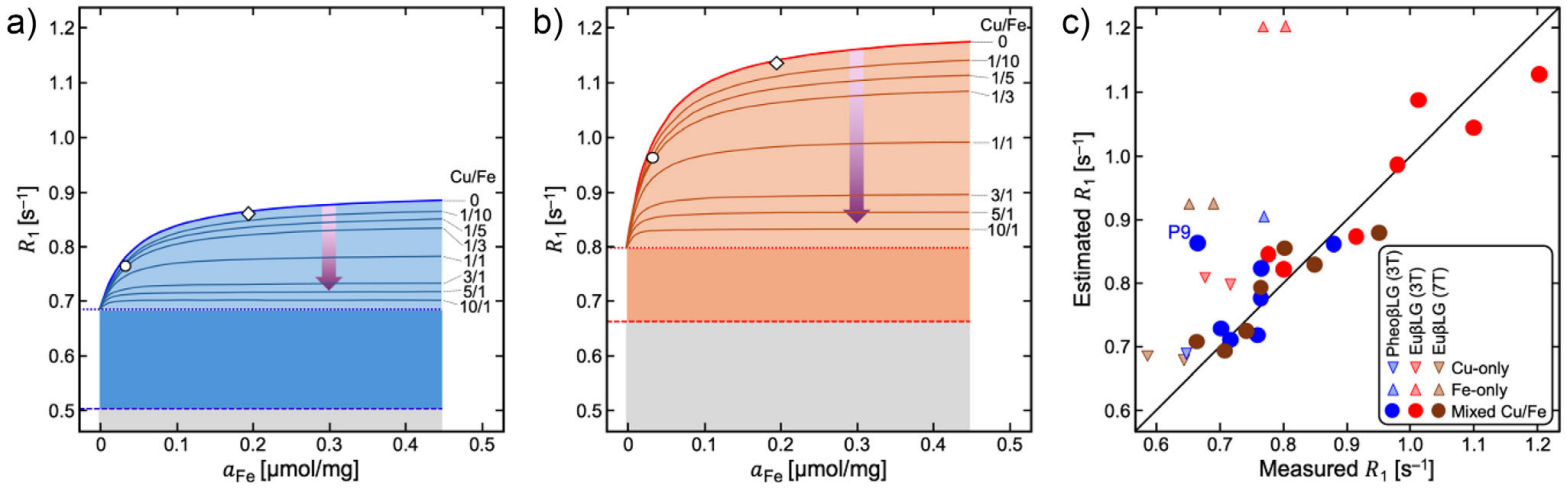
Model of the variation of *R*_1_ (at room temperature) with metal content. *R*_1_ values estimated with Eq. 7 are shown as a function of *a*_Fe_ and the Cu/Fe ratio for a) PheoβLG‐CuFe and b) EuβLG‐CuFe as well as c) in comparison with measured relaxation rates. Dashed blue and red lines indicate *R*_1,0_ in the metal‐free preparations. Dotted blue and red lines correspond to *R*_1,0_ + *R* 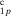, where the combined contribution from homonuclear (Cu only or Fe only) and mixed‐metal (Cu‐Fe) clusters, *R* 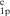, was assigned to the extrapolated *R*_1_ for $s_{\mathrm{Fe}}\to 0$ (Figure 7a) and assumed to be invariant (areas shaded in dark blue or orange. Areas shaded in light blue or orange indicate the PRE due to mononuclear Fe(III). Solid blue and red lines labeled as “0” show the *R*_1_ variation for negligible copper content. $\kappa_{\mathrm{Fe}}r_{1,\mathrm{Fe}}^{m}$ was taken from the slope of the linear‐regression result (Figure 7a) and *c*_0_ and *c*_2_ from the Langmuir model (Figure 5a). Further solid lines show *R*_1_ in mixed‐metal samples (“iso‐Cu contour lines”) for different Cu/Fe ratios of the conjugates. Arrows indicate the decreasing PRE from Fe(III) with increasing copper content. Open diamonds and circles indicate previously obtained metal concentrations in NM extracted from human *substantia nigra* and *locus coeruleus*, respectively (Table 2).

Rates computed with Eq. 7 are illustrated in Figure 8. Estimated and measured values agree quite well in mixed‐metal samples and reasonably in derivatives with only one ion type, but not in pure‐iron EuβLG preparations, where *R*_1_ is considerably overestimated.

Figures 7a and 8 illustrate that the longitudinal relaxation enhancements from clusters and mononuclear Fe(III) are of similar magnitude. As in ferritin, clustered iron is organized as antiferromagnetic Fe(III) oxy‐hydroxy aggregates,^[^ ^35^, ^38^ ^]^ in line with the XAS results. For incomplete cancellation of the magnetic sublattices (e.g., because of vacancies or insufficient exchange forces in the peripheral region), a superparamagnetic moment will result.^[^ ^56^ ^]^ A relevant contribution to $\Delta R_{1p}^{c}$ may, therefore, come from an *outer‐sphere mechanism*.^[^ ^55^ ^]^ Proton exchange between water and peripheral hydroxy groups may also contribute via an *inner‐sphere mechanism*. Considering the high abundance of aggregated ions, a substantial contribution to Δ*R*_1p_ is plausible. Both mechanisms were discussed to explain water relaxation caused by ferritin^[^ ^56^, ^57^, ^58^ ^]^ and would suggest a concentration dependence of $\Delta R_{1p}^{c}$ (e.g., a variation with the number of clusters or aggregate size). The constant term in Eq. 7 may, therefore, be an oversimplification.

Transverse relaxation showed similar trends with an enhanced contribution from clusters and a weaker *R*_2_ increase with *s*_Fe_. This supports a dominant *outer‐sphere mechanism* causing dephasing of water magnetization by diffusion in the inhomogeneous field around clusters, which is more efficient for transverse relaxation. Recent observations that static dephasing adequately described the MRI signal in ultra high‐resolution gradient and spin‐echo images of *substantia nigra* confirm this interpretation.^[^ ^21^ ^]^ The nonmonoexponential behavior suggests incomplete water exchange between mono‐ and multinuclear sites on the *T*_2_ time scale—similar to observations in porous media.^[^ ^59^ ^]^ Given the *T*_2_ range, associated diffusion lengths in the gel (order of ∼10 µm) exceed the conjugate size. Therefore, the clusters are probably buried in the conjugate core, such that water confined within these locations has low diffusion capacity to more peripheral mononuclear sites and the bulk. The increase in the faster‐relaxing component in the presence of excess iron would confirm this assumption if added metal preferentially populates mononuclear sites without accessing the clusters. This is similar to natural NMs, where treatment with strong chelating agents does not remove aggregated iron but decreases the Fe(III) EPR signal, suggesting that iron does not migrate between both positions.^[^ ^6^ ^]^

Interestingly, deviations between calculated and experimental *R*_1_ values were largest for Fe‐only conjugates (Figure 8c), particularly for eumelanins. Probably, the compensation of magnetic moments of the antiferromagnetic sublattices is less effective in heteronuclear clusters, supporting more efficient proton relaxation. Alternatively, simultaneous presence of copper and iron during the reaction could accelerate DA oxidation resulting in smaller aggregates. This might modulate the diffusion characteristics and accessibility of metal sites and, thereby, the relaxation dynamics. We note that the parameters in Eq. 7 were obtained from fits to data from mixed‐metal preparations due to their more consistent variation of the composition (i.e., potentially smaller contributions to $\Delta R_{1p}^{c}$ would not be captured). Similar mechanisms might be responsible for deviations from the general trends that were observed for mixed‐metal samples with highest iron content.

The overall small variation of Δχ (Figure 7c) rules out that susceptibility is dominated by paramagnetic mononuclear ions. Neuromelanin‐iron clusters are thought to have a similar structure as the iron core in ferritin, but are smaller, with a range of blocking temperatures and no well‐defined grain dimension.^[^ ^60^ ^]^ Estimating the susceptibility contribution of decoupled spins in superparamagnetic subgrains is not trivial as it depends on the specific architecture of the antiferromagnetic sublattices.^[^ ^61^ ^]^ The slight decay of Δχ with p suggests an inverse relationship with grain size and a shift toward larger subgrains with increasing metal content.

### Structural Model for Neuromelanin–Protein Conjugates

**Figure 9:**
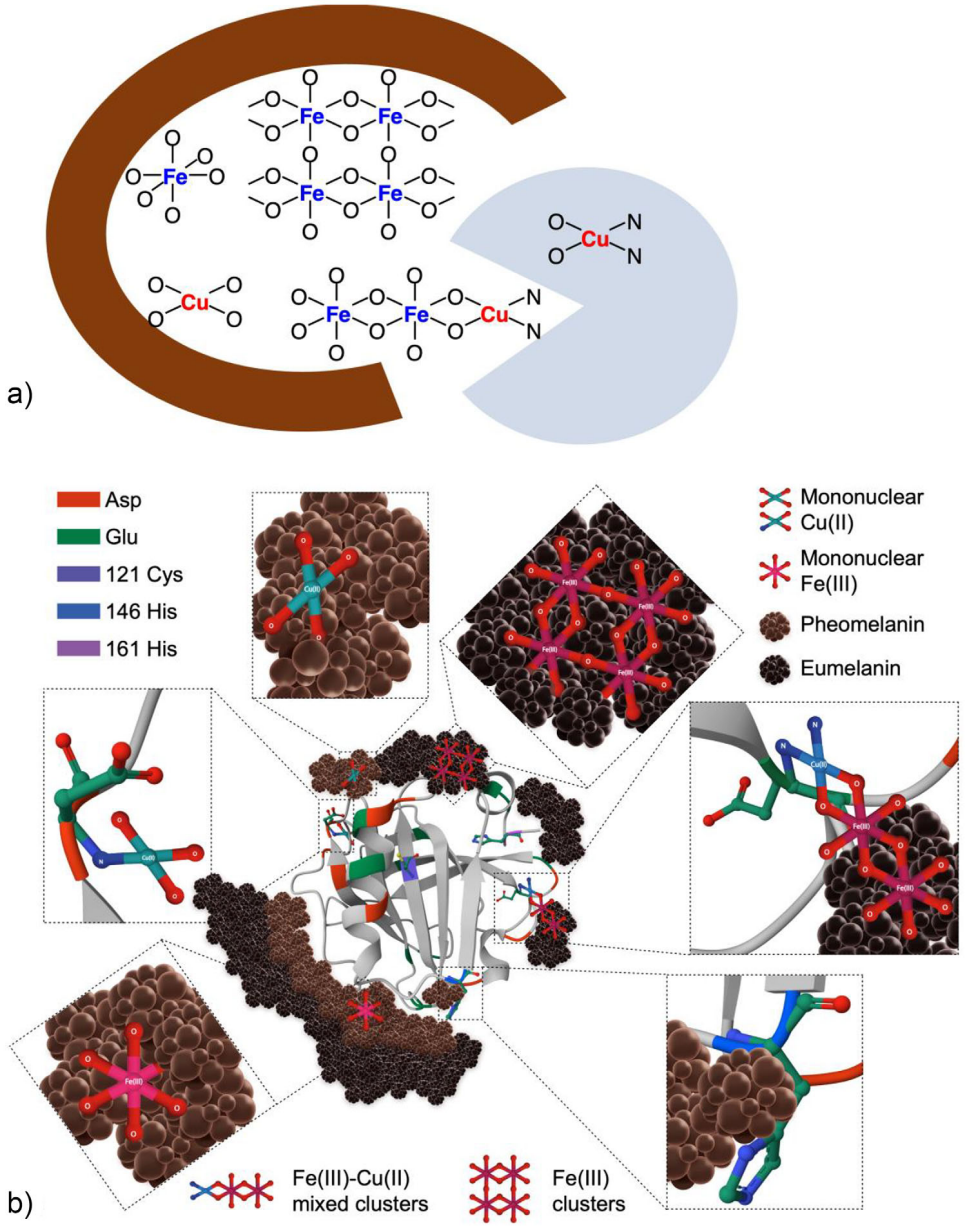
Illustration of the assumed metal binding sites in melanin–protein conjugates. a) Schematic representation of the various types of binding sites and metal environments in the melanic (red) and protein (blue) parts of PheoβLG‐CuFe and EuβLG‐CuFe conjugates. b) Structural model of melanin–βLG conjugates showing the pheomelanic and eumelanic components surrounding the protein core and the distribution of various mononuclear and multinuclear metal sites in the melanic and protein components. For obvious reasons the size of the clusters is kept at a minimum.

Integration of all results leads to the following hypothetical structural model: The protein component contains Cys121 and His161 as potential metal binding residues (unmodified by DA‐quinones) and, additionally, the *N*‐terminal fragment for Cu(II). The cysteine is at the bottom of a calyx surrounded by nonpolar amino acids, which limits access to hydrated metal ions. Consistently, XAS and EPR do not indicate binding of Fe(III) or Cu(II) to sulfur donors. Some of the several Asp and Glu carboxylate side chains in non‐structured loops of βLG may represent anchoring groups for Cu(II), allowing binding to amide groups in the backbone, in line with ∼20% Cu(II)‐N local environments in PheoβLG‐CuFe‐1/10. The catecholate groups of the melanic components are main binding sites for Fe(III) and anchors for Fe clusters. Particularly at high *a*_Cu_, Cu(II) will compete for these sites, consistent with the relaxation results. For PheoβLG derivatives, which better mimic natural NMs, initial events during synthesis involve the reaction of cysteine with DA‐quinone, generating cysteinyl‐DA.^[^ ^28^ ^]^ This may then i) participate in the growing melanin chain,^[^ ^43^, ^46^ ^]^ constituting the pheomelanin coat around the protein core;^[^ ^8^, ^62^ ^]^ ii) undergo oxidation to cysteinyl‐DA‐quinone, competing with DA‐quinone for nucleophilic groups; iii) chelate Fe(III) or Cu(II) through catechol groups; or iv) evolve to benzothiazine moieties.^[^ ^63^ ^]^ Participation of benzothiazine residues in the chelation appears unlikely as metal–sulfur bonds were not detected. Primary binding sites for Cu(II) are then located within the βLG residue (Figure 9a), where it may form mixed‐metal clusters with Fe(III) on the melanin surface but also occupy mononuclear sites binding to nitrogen donors. Excess Cu(II) will also bind to catecholate groups of melanin, partly replacing Fe(III) and shifting it to nearby aggregated clusters. This explains the apparently reduced relaxivity produced by Cu(II). A structural model encompassing the key aspects of the present melanin‐βLG conjugates is shown in Figure 9b. Conjugates that better reflect the composition of *locus coeruleus* NM regarding metal‐ion composition are PheoBLG‐CuFe‐1/3 and PheoBLG‐CuFe‐1/5 (Table 1).

## Conclusion

Neuromelanin biosynthesis involves iron and probably—if available—copper in early steps, and natural NM is always bound to iron and other metals.^[^ ^1^ ^]^ Unless saturated in pathological conditions, NM retains a protective buffering capacity for age‐related iron accumulation/dysmetabolism.^[^ ^6^, ^45^ ^]^ Indeed, aging is the major risk factor for neurodegenerative disorders, including PD and AD.^[^ ^64^ ^]^ Neuromelanin‐MRI has been proposed as a biomarker for *substantia nigra* and *locus coeruleus* degeneration to show the conversion from prodromal to clinical PD and follow disease progression and noradrenergic dysfunction in AD.^[^ ^13^, ^16^, ^65^ ^]^ The NM‐MRI signal in the *substantia nigra* appears to be related to DA release, making it a potential functional marker in the nigrostriatal system.^[^ ^66^ ^]^ An imaging method that reveals presymptomatic NM loss would be crucial for developing neurorescuing treatments and evaluating pharmacological interventions targeting dopaminergic and noradrenergic neurotransmission. Ideally, this should include information on metals bound to NM.

This work studies models that mimic NM to characterize binding sites in melanin‐protein conjugates and investigate proton relaxivity. Neuromelanin's ability to form paramagnetic complexes with metals is key to its MRI detection, facilitated by enhanced relaxation.^[^ ^12^ ^]^ Since the PRE is not accessible in vivo, systematic investigations in model systems with variable Cu/Fe ratios are needed. These soluble melanin‐βLG derivatives are also suitable for cell culture and animal experiments to clarify pathological mechanisms of neuronal death involving NM.

Water proton relaxation and magnetic susceptibility are *sensitive* indicators of NM as shown here as well as previously by demonstrating correlations with NM concentration in *substantia nigra* ^[^ ^66^ ^]^ and with total iron (but *not* ferritin) content in *substantia nigra* ^[^ ^67^ ^]^ and dopaminergic neurons.^[^ ^21^, ^22^ ^]^ There is also initial evidence that NM‐sensitive MRI reveals abnormality in *substantia nigra* and *locus coeruleus* of Parkinson's patients^[^ ^68^, ^69^ ^]^ and in *locus coeruleus* of Alzheimer's patients^[^ ^15^ ^]^ already in presymptomatic phases, which worsens as the disease progresses. Methods that achieve presymptomatic diagnosis would support the development of neurorescuing treatments that at least slow the disease progression.

This work is a first step toward optimized methods with improved sensitivity and, ideally, specificity that can detect early loss of NM‐containing neurons before symptoms appear, at a time when compensatory mechanisms are still in place and diseases are clinically “silent.” Use of synthetic melanins allowed a systematic adjustment of the metal content bound to NM to evaluate associated effects on MRI contrast, including the role of copper, which was not considered previously. The results can be used to model MRI contrast as a function of NM content and composition for optimizing imaging protocols. Remarkably, all MRI parameters exhibited nonlinear behavior with respect to the iron content reflecting the complexity of the NM structure, composition, metal binding, and related magnetic properties. For example, *R*_1_ increased by ∼50% and ∼70% for metal contents typical of *locus coeruleus* and *substantia nigra*, respectively (Figure 8), which is easy to differentiate. However, doubling the nigral iron content would additionally change *R*_1_ by less than 5%. Taken together, MRI primarily assesses average cellular NM concentrations, with only little information on the distribution of conjugate sizes and composition. Nevertheless, there is limited *specificity*, considering that *R*_1_ contained similar contributions from mono‐ and multinuclear iron, while the impact from clusters became stronger for *R*_2_—and probably even more for $R_{2}^{*}$ ^[^ ^21^ ^]^—and dominant for Δχ. Multiparametric imaging of *R*_1_, $R_{2}^{*}$ and Δχ could, therefore, provide additional information. It can be performed with gradient‐echo techniques at high spatial resolution^[^ ^70^ ^]^ as required for evaluating *substantia nigra* and *locus coeruleus*, and also simultaneously in a single MRI sequence.^[^ ^71^ ^]^ These MRI methods are reasonably fast, inexpensive (compared to the disease burden and related cost) and safe. They are suitable as mass screening procedures to evaluate subjects at risk of PD and related disorders (e.g., multiple system atrophy, progressive supranuclear palsy, corticobasal degeneration) and AD for genetic, occupational, or behavioral factors.

## Supporting Information

Synthesis of melanin‐βLG conjugates. Characterization of the conjugates. Preparation of polyacrylamide gel phantoms. NMR of human NM isolated from *substantia nigra*. EPR experiments. XAS measurements. MRI acquisitions. Langmuir model for binding of EPR‐active metal ions. Statistical analysis. Supporting data. Supporting tables. Supporting figures. The authors have cited additional references within the Supporting Information.^[^ ^72^, ^73^, ^74^, ^75^, ^76^, ^77^, ^78^, ^79^, ^80^, ^81^, ^82^, ^83^, ^84^, ^85^, ^86^, ^87^, ^88^, ^89^, ^90^ ^]^

## Conflict of Interests

The authors declare no conflict of interest.

## Supporting information



Supporting Information

## Data Availability

The data that support the findings of this study are available from the corresponding author upon reasonable request.
