# Supplementary material for: Modeling Midbrain and Brainstem Neuromelanins to Characterize Metal Binding and Associated MRI Contrast in Parkinson's and Alzheimer's Diseases
Source: Angew Chem Int Ed Engl. 2025 Sep 19;64(41):e202509102. doi: 10.1002/anie.202509102 (PMC12501727; doi:10.1002/anie.202509102)
Supplement: Supplementary file 1 — Supporting Information [file ANIE-64-e202509102-s001.pdf]

Supporting Information

Modeling Midbrain and Brainstem Neuromelanins to Characterize Metal Binding and Associated MRI Contrast in Parkinson's and Alzheimer's Diseases

N. Wallstein,[1] A. Capucciati,[2,3] A. Pöppl,[4] C. S. Schnohr,[4] M. Sturini,[2] A. Pampel,[1] C. Jäger,[5,6] L. Zecca,[7] F. A. Zucca,[7] E. Monzani,[2] L. Casella,[2] H. E. Möller[1,4,]*

[1] NMR Methods & Development Group, Max Planck Institute for Human Cognitive and Brain Sciences, Stephanstraße 1A, 04103 Leipzig (Germany)

[2] Department of Chemistry, University of Pavia, Via Taramelli 12, 27100 Pavia (Italy)

[3] Fondazione Pezzoli per la Malattia di Parkinson, Via Gianfranco Zuretti 35, 20125 Milan (Italy)

[4] Felix Bloch Institute for Solid State Physics, Leipzig University, Linnéstraße 5, 04103 Leipzig (Germany)

[5] Department of Neurophysics, Max Planck Institute for Human Cognitive and Brain Sciences, Stephanstraße 1A, 04103 Leipzig (Germany)

[6] Paul Flechsig Institute – Centre of Neuropathology and Brain Research, Leipzig University, Liebigstraße 19, 04103 Leipzig (Germany)

[7] Institute of Biomedical Technologies, National Research Council of Italy, Via Cervi 93, 20054 Segrate (Milan) (Italy)

* E-mail: moeller@cbs.mpg.de

TABLE OF CONTENTS

Abbreviations 4

Mathematical Symbols 4

Experimental Section 6

- **Synthesis of Melanin-βLG Conjugates** 6
- **Characterization of the Conjugates** 6
- **Preparation of Polyacrylamide Gel Phantoms** 6
- **^{1}H NMR of Human NM Isolated from *Substantia Nigra*** 7
- **EPR Experiments** 7
- **XAS Measurements** 8
- **MRI Acquisitions** 8
- **Langmuir Model for Binding of EPR-Active Metal Ions** 10
- **Statistical Analysis** 12

Supporting Data 13

Supporting Tables 14

- **Table S1.** βLG fragments of both conjugates that were not identified after digestion with combined trypsin and pepsin 14
- **Table S2.** X-band EPR acquisition parameters 14
- **Table S3.** Results of LCA fits of Cu K-edge XANES spectra of PheoβLG-Cu-5% and PheoβLG-CuFe-1/10 15
- **Table S4.** Results of proton relaxation and susceptibility measurements at room temperature. 16

Supporting Figures 17

- **Figure S1.** X-band EPR spectra of melanin-protein conjugates recorded at ~300 K 17
- **Figure S2.** Variation of the EPR-active Cu/Fe ratio (measured at 10 K) with the total Cu/Fe ratio 17
- **Figure S3.** Dependencies of the EPR signals of Fe(III) and Cu(II) (measured at 10 k) on total metal contents 18

Figure S4. Lineweaver-Burke plot of the variation of $1/s_{\mathrm{Fe}}$ (measured at 10 K) with $1/a_{\mathrm{Fe}}$ without competition for binding sites 19

Figure S5. Quantitative analysis of the XAS data 20

Figure S6. Water proton T_1 maps measured at 3 T and room temperature 21

Figure S7. Comparison of water proton T_1 in EuβLG samples at 3 T and 7 T (both at room temperature) 21

Figure S8. Proton longitudinal relaxation rates at 3 T and room temperature in dependence of the metal content 22

Figure S9. Proton transverse relaxation rates at 3 T and room temperature in dependence of the metal content 22

Abbreviations

3D = three-dimensional; a.u. = arbitrary units; BIR = B_1^+-insensitive rotation; BSA = bovine serum albumin; CPMG = Carr-Purcell-Meiboom-Gill; DA = dopamine; EPR = electron paramagnetic resonance; Eu = eumelanin; EXAFS = extended X-ray absorption fine structure; FT = Fourier transform; ICP-OES = inductively coupled plasma optical emission spectroscopy; LCA = linear combination analysis; LC-MS = liquid chromatography-mass spectrometry; MRI = magnetic resonance imaging; NM = neuromelanin, NM-MRI = neuromelanin-sensitive MRI; NMR = nuclear magnetic resonance; NN = nearest neighbor; PD = Parkinson's disease, PRE = paramagnetic relaxation enhancement; Pheo = pheomelanin; ROI = region of interest; SD = standard deviation; SEM = standard error of the mean; XANES = X-ray absorption near edge structure; XAS = X-ray absorption spectroscopy; βLG = β-lactoglobulin.

Mathematical Symbols

A_{M}^{m}: total number of mononuclear binding sites for metal M;
a_{M}: total amount of M in the in the melanin-protein conjugate in μmol/mg;
$\tilde{a}_{\mathrm{M}}$: total amount of M in the in the melanin-protein conjugate in μg/mg;
a_{M}^{k}: amount of metal M at binding site k in μmol/mg;
$a_{\mathrm{M,aq}}$: concentration of metal ion M;
B_1^+: radiofrequency transmit magnetic field;
c_0, c_1, c_2: linear fitting parameters;
g: effective g-factor;
$g_{||}$: g-tensor hyperfine component parallel to the magnetic field;
K_{M}: equilibrium constant for metal M;
$K_{m,\mathrm{M}}$: binding constant for metal M at mononuclear sites;
$K'_{m,\mathrm{M}}$: binding constant for metal M at mononuclear sites normalized by K_{M};
k: photoelectron wave number;
$k \in \{m, c\}$: index of the binding type for metal ions;
$\mathrm{M} \in \{\mathrm{Cu, Fe}\}$: index of the metal ion type;
n: number of samples;
$\wp$: effective concentration of Bohr magnetons;
R: R-factor;
R_1: longitudinal relaxation rate;
$R_{1,0}$: longitudinal relaxation rate of the metal-free melanin-βLG preparation;
ΔR_{1p}: metal ion-induced longitudinal relaxation enhancement;
ΔR_{1p}^{c}: longitudinal relaxation enhancement due to metal clusters;
ΔR_{1p}^{m}: longitudinal relaxation enhancement due to mononuclear metal centers;
R_2: transverse relaxation rate;
R_2^*: effective transverse relaxation rate;
$R_{2,0}$: transverse relaxation rate of the metal-free melanin-βLG preparation;
R_{2p}^{c}: transverse relaxation enhancement due to metal clusters;
R_2^{mono}: monoexponential transverse relaxation rate;
r^2: squared Pearson correlation coefficient;

$r_{1,\mathrm{M}}^{k}$:	longitudinal relaxivity of ion type M at binding site k;
$r_{2,\mathrm{M}}^{k}$:	transverse relaxivity of ion type M at binding site k;
S:	total electronic spin quantum number;
s_{M}:	EPR signal area or amplitude of metal ion M;
T_1:	longitudinal relaxation time;
T_2:	transverse relaxation time;
T_2^{mono}:	monoexponential transverse relaxation time;
TE:	echo time;
TR:	repetition time;
κ_{M}:	scaling factor to convert EPR signal amplitudes into metal content;
τ:	evolution time;
φ:	proportionality factor, slope;
$\Delta\chi$:	bulk magnetic susceptibility.

Experimental Section

Synthesis of Melanin-βLG Conjugates

The model NMs were synthesized by oxidation of DA in the presence (Pheo) or absence (Eu) of l-cysteine, βLG, and variable amounts of iron and copper ions. The DA:βLG ratio was 1:2 (w/w). The procedures to prepare melanin-βLG conjugates and their derivatives containing iron or copper were as reported elsewhere.[44, 46] Dopamine and metal salts $Fe(NH_4)_2(SO_4)_2 \cdot 6H_2O$ and $Cu(NO_3)_2 \cdot 3H_2O$ were purchased from Merck KGaA (Darmstadt, Germany). β-Lactoglobulin was purchased from Sigma-Aldrich (St. Louis, MO, USA) in polymorphic forms A and B that differ by substitutions of aspartate for glycine at position 64 and valine for alanine at position 118. The DA oxidative polymerization was controlled to obtain samples with reasonable water solubility (final melanin/protein ratio ~1:1), which allowed their thorough characterization.

We note that native βLG was used instead of its fibrillated form to reduce the sample heterogeneity. This generates melanin-protein conjugates more adherent to the core of NM.[46] Model NMs obtained from DA, βLG and cysteine mimic NM in *substantia nigra* neurons.[3] Precursor catecholamines in NM of the noradrenergic neurons of *locus coeruleus* are mainly both DA and norepinephrine.[72] However, in synthetic melanin-protein conjugates prepared with norepinephrine, it is more difficult to adjust the amount of metal ion reagents to obtain samples with a final Cu/Fe ratio approximating the value in human NM.[44]

Characterization of the Conjugates

The initial sample characterization included determinations of the protein fraction and metal content, high-resolution ^{1}H NMR in D_2O and the identification of attachment sites of DA quinones to βLG through LC-MS. The protein content was determined after complete acid hydrolysis and NMR quantification of selected amino acids,[43] and the iron and copper content by ICP-OES (iCAP 7400, Thermo Fisher Scientific, Waltham, MA, USA) after acidic digestion.[46]

Solutions for NMR investigations were prepared by treating the lyophilized samples (~1 mg/ml) with D_2O under stirring and protection from light for two days. The solutions/suspensions were allowed to settle, and ^{1}H NMR spectra were recorded at room temperature on the supernatants on an AVANCE 400 spectrometer (Bruker BioSpin, Ettlingen, Germany), operating at a proton frequency of 400.13 MHz (accumulation of >2,000 scans).

Proteolysis of the melanin-βLG conjugates was carried out after denaturation in the presence of a reducing agent.[44]

Preparation of Polyacrylamide Gel Phantoms

To roughly adjust the relaxation rates to a typical range in brain tissue *in vivo*, phantoms containing a polyacrylamide gel matrix were used for MRI experiments. The gel was prepared according to the protocol for polyacrylamide gel electrophoresis, but without sodium dodecyl sulphate.[41] Semisolid tissue proteins were modelled by adding bovine serum albumin (BSA) before the acrylamide polymerization. 10% (w/v) BSA (Sigma-Aldrich A4503) in 10%

acrylamide/bis-acrylamide mixture (37.5:1) in 0.4 m Tris-HCl (pH 7.4) yielded a semisolid matrix. Appropriate quantities (0.5 mg/ml) of the melanin preparations or βLG (used as reference) were suspended rapidly and uniformly in the base gel while avoiding air-bubble formation and sedimentation. The preparations (3 ml each) were placed in 7-ml plastic tubes (16-mm inner diameter), sandwiching them between plugs of polyacrylamide to minimize susceptibility gradients at the boundaries. The polyacrylamide plug at the tube tip was allowed to set prior to loading the preparation, whereas the second plug was loaded after the preparation had set.

^{1}H NMR of Human NM Isolated from *Substantia Nigra*

An unpublished NMR spectrum of NM isolated from human *substantia nigra* tissue in previous work[25] was available for comparison with the spectra of synthetic NM. This study had been approved by the Institutional Review Board of Institute of Biomedical Technologies – National Research Council of Italy (Segrate, Milan, Italy) and carried out in compliance with the Policy of the National Research Council of Italy. Written informed consent for using brain samples for research purposes was obtained from closest relatives and is kept at the Section of Legal Medicine and Insurances, Department of Human Morphology and Biomedical Sciences, University of Milan. The ^{1}H NMR spectrum was acquired on an AVANCE 400 spectrometer (Bruker BioSpin) at 293 K (accumulation of 8,192 scans), using the standard Bruker *p3919gp* pulse program. A 150-ms delay was applied for binomial water-sginal suppression with WATERGATE (WATER suppression by GrAdient-Tailored Excitation). The NM pigment had been isolated from pooled *substantia nigra* tissues as previously reported. For details on the tissue treatments, refer to Zecca et al.[1] and Engelen et al.[25] Due to the insolubility in water, the human NM solution was prepared by gently stirring approximately 1.0 mg of the pigment in 0.7 mL of 99.8% DMSO-d_6 for 48 hours to ensure proper dissolution of the NM fraction of lower molecular mass.[25] Prior to data acquisition, the undissolved solid was removed by centrifugation.

EPR Experiments

Small amounts (2–3 mg) of selected lyophilized melanin preparations were examined by EPR as dry powders. Continuous-wave spectra (8,192 linearly spaced points) were recorded as first derivatives on an X-band spectrometer (EMXmicro; Bruker BioSpin) at 10 K, 60 K and room temperature and processed with the EasySpin toolbox (v5.2.33; https://easyspin.org/).[73] Acquisition parameters are summarized in Table S2. A spectrum of the 'empty cavity' was always subtracted to avoid possibly interfering signal contributions. The signal intensities were divided by the receiver gain and the sample mass to ensure comparability of the acquisitions. The relative amounts of paramagnetic ions were estimated from the peak height of the signal at 95–235 mT for Fe(III) and by numerical integration of the spectra twice in the range of 255–365 mT for Cu(II).

XAS Measurements

Three PheoβLG samples (Cu-5%, Fe-10% and CuFe-1/10) were investigated by XAS. The refrigerated dry powders (14–18 mg) were warmed up, mixed with ~19 mg of ultra-high-purity graphite powder (ThermoFisher Scientific, Waltham, MA, USA) and manually ground for 15 min in an agate mortar. The mixture was then pressed into 1-mm thick, 5-mm diameter pellets, which were stored in a freezer until the measurements were taken. Graphite-diluted powder pellets of Fe_2O_3, Cu_2O, CuO (ThermoFisher) and CuS (Sigma-Aldrich) were prepared as standards.

XAS measurements were performed at beamline P65 of PETRA III at DESY, Germany.[74] Spectra were recorded at the Fe and Cu K-edge (7.112 and 8.979 keV, respectively) in transmission mode at 10 K. A Fe or Cu foil was measured simultaneously to enable precise energy alignment of the different spectra. Series of short XANES scans (60 s) and normal XANES + EXAFS scans (300 s) were performed to confirm that the samples did not exhibit radiation-induced changes on a minutes-to-hours time scale. At the Fe K-edge, the step height was ~0.9, and single scans were used in the analysis. At the Cu K-edge, the step height was 0.1–0.2, and ten scans were averaged.

The data were analyzed using the IFEFFIT code and ATHENA and ARTEMIS interfaces.[75, 76] Phase shifts and scattering amplitudes were calculated using FEFF9.[77] The XANES of the normalized spectra was analyzed by LCA using the measured standards. Spectra of FeO, FeS, FeS_2, Fe_3N, Cu_2S, $Cu(NO_3)_2 \cdot 3H_2O$ and Cu_3N were taken as additional standards from the MDR XAFS Database (see Supporting Data).[78,79] The fits were performed from –20 to 30 eV around the half height of the edge. Estimated overall uncertainties included different combinations of standards, systematic variations of fitting range and fit settings, and uncertainties of the fit.

The EXAFS was Fourier-transformed over a photoelectron wave-number range of k = 3–11 $Å^{-1}$ using a Hanning window (tapering 2 $Å^{-1}$). The Fourier transforms were analyzed with a path-fitting approach using either Fe_2O_3 or CuO as basic structural model and adding N, S, Fe or Cu to the local environment of the absorbing atom. Fitting was performed in radial space with multiple k-weights of 2,3. For each scattering path, the average interatomic distance (bond length) and the variance of the distance distribution (disorder) were fitted. The amplitude reduction factor and the difference of the threshold energy were set to average values for all samples measured at a given edge. Estimated overall uncertainties included systematic variations of the window parameters for FT, k-weights and fitting range, and uncertainties of the fit.

MRI Acquisitions

Most MRI experiments were performed at 3 T on a MAGNETOM Prisma^{fit}, Skyra^{fit} or Skyra Connectom A (Siemens Healthineers, Erlangen, Germany) depending on availability. For first measurements, all suspensions of the same batch (i.e., all PheoβLG and, separately, all EuβLG samples) were examined simultaneously in a custom-made sample holder with a 32-channel receive head coil and transmission via the body coil. The holder was printed on an Objet Eden260VS (Stratasys, Eden Prairie, MN, USA) from photopolymer material (Biocompatible

Clear MED610; Stratasys) and positioned inside a cylindrical container filled with 1.5% low-melting agarose in phosphate-buffered saline (pH 7.4) with 0.1% gadopentetate dimeglumine (Magnevist®, Bayer Pharma, Berlin, Germany). Room-temperature relaxation measurements were obtained from a single, 2-mm thick slice, oriented perpendicular to the cylinder axis (in-plane nominal resolution 0.5×0.5 mm^2). R_1 was measured with an inversion-recovery turbo spin echo sequence (33 evolution times, τ = 0.025–10 s; repetition time, TR = 12 s; echo time, TE = 14 ms; turbo factor 7; bandwidth 130 Hz/pixel) and R_2 with a Carr-Purcell-Meiboom-Gill (CPMG) sequence (TR = 10 s; 32 echoes, TE = 20–640 ms for Pheo and 13–416 ms for Eu; bandwidth 260 Hz/pixel). Quantitative susceptibility mapping was performed with a three-dimensional (3D) spoiled gradient-echo sequence (nominal resolution 1×1×1 mm^3; flip angle 35°; TR = 50 ms; 12 echoes, TE = 3.54–44.48 ms; acceleration factor 2, partial-Fourier factor 7/8; bandwidth 600 Hz/pixel).

Additional, more extensive 3T measurements without spatial encoding were performed in the EuβLG samples with a custom-made Helmholtz coil.[80] An appropriate quantity of the melanin/gel preparation was placed in a 3D-printed spherical container (24-mm inner diameter), and the surrounding space was filled with Fomblin® (Solvay Solexis, Bollate, Italy) to achieve sufficient homogeneity. R_1 was measured with saturation-recovery (5-ms adiabatic BIR-4 pulses; 55 evolution times, τ = 0.01–6.02 s; TR = 12 s; bandwidth 5 kHz) and R_2 with a CPMG sequence (2.5-ms BIR-4 excitation and 6-ms BIR-4 refocusing pulses; TR = 10 s; 64 echoes; TE = 0.02–1.28 s; bandwidth 5 kHz). Contributions from stimulated echoes were suppressed by crusher gradients (duration 4 ms) with alternating polarity and decreasing amplitude around each refocusing pulse.[81]

In the EuβLG samples, non-localized saturation-recovery measurements (acquisition parameters as above) were also performed at 7 T (MAGNETOM Terra; Siemens Healthineers) with a miniCP coil.[82]

Analysis scripts for MRI were implemented in Matlab (R2020b; MathWorks, Natick, MA, USA). For multiple-sample experiments, a circular region of interest (ROI) of 80±2 voxels was defined for each sample and the results from single-voxel fits averaged. Error estimates either refer to one standard deviation (SD) across the ROI or to the SD from multiple measurements. Unless otherwise stated, relaxation rates were obtained by non-linear least-squares fitting to a monoexponential signal recovery or echo-amplitude decay using the lsqnonlin function.

To analyze the influence of the metal content on proton relaxation, R_1 was decomposed into a contribution $R_{1,0}$ measured in the metal-free melanin-βLG preparation and an additional enhancement term due to ions bound to the conjugates:

$$R_1 = R_{1,0} + \Delta R_{1p}. \tag{S1}$$

The existence of multiple binding sites is well established for Fe(III) and probably also applies to Cu(II).[44] Therefore, we assumed contributions from clusters and mononuclear binding sites (indicated by superscript $k \in \{c, m\}$) to the enhancement term:

$$\Delta R_{1p} = \Delta R_{1p}^{c} + \Delta R_{1p}^{m} = \Delta R_{1p}^{c} + \sum\nolimits_{\mathrm{M}} a_{\mathrm{M}}^{m}\, r_{1,\mathrm{M}}^{m}. \quad \text{(S2)}$$

ΔR_{1p}^{m} was modeled as classical PRE due to two species M ∈ {Fe, Cu} with "concentrations" a_{M}^{m} in μmol/mg and relaxivities $r_{1,\mathrm{M}}^{m}$ in s^{-1}/(μmol/mg). Similarly, the transverse relaxation rate was approximated as

$$R_{2}^{\mathrm{mono}} = R_{2,0} + \Delta R_{2p}^{c} + \sum\nolimits_{\mathrm{M}} a_{\mathrm{M}}^{m}\, r_{2,\mathrm{M}}^{m}. \quad \text{(S3)}$$

Details of the steps to extract susceptibilities are described elsewhere.[83] Briefly, the multi-receive-channel data of the 10th echo (TE ≈ 37 ms) were combined without phase singularities using an adaptive-combine algorithm. After Laplacian phase unwrapping[84] and background-field removal,[85] maps of $\Delta\chi$ were reconstructed using the iLSQR method implemented in the STI suite.[86]

Langmuir Model for Binding of EPR-Active Metal Ions

In the following, we develop a simplifying model for the amounts of *EPR-active*, mononuclear Fe(III) and Cu(II) ions (bound to the melanin-βLG conjugate and available in arbitrary units from the scaled EPR spectra) as a function of the total contents a_{M} (measured by ICP-OES in μmol/mg). The model does not attempt to provide a quantitative description of *EPR-silent* metal ions aggregated in multinuclear clusters. However, we assume that the population of the different binding sites occurs independently of each other according to their specific affinities, which is supported by experimental studies of the iron-binding characteristics of human neuromelanin and synthetic dopamine melanins.[36, 87]

Considering previous results obtained under similar preparation conditions,[44, 46] the amounts (in μmol/mg) $a_{\mathrm{M,aq}}$ of substrate metal ions and corresponding amounts coupled in multinuclear clusters, a_{M}^{c}, and bound at isolated mononuclear sites, a_{M}^{m}, are typically characterized by $a_{\mathrm{M,aq}} \gg a_{\mathrm{M}}^{c} + a_{\mathrm{M}}^{m} \equiv a_{\mathrm{M}}$. As a simplification, we describe the relation between $a_{\mathrm{M,aq}}$ and the total amount of metal a_{M} by an apparent equilibrium constant,

$$K_{\mathrm{M}} = \frac{a_{\mathrm{M}}}{a_{\mathrm{M,aq}}}, \quad \text{(S4)}$$

and estimate a_{M}^{m} from the EPR signal (Eq. 6). While this is at best a rough approximation, it is supported to some extent by studies of the iron-binding characteristics.[36, 87] They suggest saturation of high-affinity binding sites assigned to EPR-silent clusters at high substrate concentrations. The amounts of aggregated ions in different samples might then be roughly constant, and K_{M} would primarily reflect low-affinity binding sites, whose population appears to be in a linear regime for most compositions investigated in our work.

Iron(III) is known to bind to oxygen atoms of the melanic catechol moieties at distinct sites,[8, 28, 35] whereas it was recently proposed that Cu(II) binds, at least at low a_{Cu}, to the protein in mixed oxygen/nitrogen coordination.[44] For a more general description, we consider the

additional possibility that both metals bind to equivalent sites and model their occupancy according to competitive monolayer adsorption:[88, 89]

$$a_{\mathrm{M}}^{m}=\frac{A_{\mathrm{M}}^{m}K_{m,\mathrm{M}}a_{\mathrm{M,aq}}}{1+\sum_{\mathrm{M}}K_{m,\mathrm{M}}a_{\mathrm{M,aq}}}=\frac{A_{\mathrm{M}}^{m}K'_{m,\mathrm{M}}a_{\mathrm{M}}}{1+\sum_{\mathrm{M}}K'_{m,\mathrm{M}}a_{\mathrm{M}}} \tag{S5}$$

with

$$K'_{m,\mathrm{M}}\equiv\frac{K_{m,\mathrm{M}}}{K_{\mathrm{M}}}. \tag{S6}$$

A_{M}^{m} denotes the total number of mononuclear binding sites (occupied plus unoccupied) and the reciprocal binding constant $1/K_{m,\mathrm{M}}$ equals the substrate concentration required for half-saturation (equivalent to the Michaelis constant). It is advantageous to rewrite Eq. S5 for iron and consider two limiting cases, *(i)* a much higher binding affinity for Fe(III) than for Cu(II) and *(ii)* a high metal content:

$$a_{\mathrm{Fe}}^{m}=\frac{\kappa_{\mathrm{Fe}}a_{\mathrm{Fe}}}{c_1+c_0a_{\mathrm{Fe}}+c_2a_{\mathrm{Cu}}}\approx\begin{cases}\dfrac{\kappa_{\mathrm{Fe}}a_{\mathrm{Fe}}}{c_1+c_0a_{\mathrm{Fe}}} & \text{if}\quad c_1+c_0a_{\mathrm{Fe}}\gg c_2a_{\mathrm{Cu}}\\ \dfrac{\kappa_{\mathrm{Fe}}a_{\mathrm{Fe}}}{c_0a_{\mathrm{Fe}}+c_2a_{\mathrm{Cu}}} & \text{if}\quad c_0a_{\mathrm{Fe}}+c_2a_{\mathrm{Cu}}\gg c_1\end{cases} \tag{S7}$$

with

$$c_0\equiv\frac{\kappa_{\mathrm{Fe}}}{A_{\mathrm{Fe}}^{m}},\quad c_1\equiv\frac{c_0}{K'_{m,\mathrm{Fe}}}\quad\text{and}\quad c_2\equiv\frac{K'_{m,\mathrm{Cu}}}{K'_{m,\mathrm{Fe}}}c_0. \tag{S8}$$

Linearization[90] and insertion of Eq. 6 leads to:

$$\frac{1}{s_{\mathrm{Fe}}}\approx\begin{cases}c_1\cdot\dfrac{1}{a_{\mathrm{Fe}}}+c_0 & \text{if}\quad c_1+c_0a_{\mathrm{Fe}}\gg c_2a_{\mathrm{Cu}}\\ c_2\cdot\dfrac{a_{\mathrm{Cu}}}{a_{\mathrm{Fe}}}+c_0 & \text{if}\quad c_0a_{\mathrm{Fe}}+c_2a_{\mathrm{Cu}}\gg c_1\end{cases}. \tag{S9}$$

An equivalent expression for Cu(II) binding may be conveniently written as:

$$a_{\mathrm{Cu}}^{m}=\frac{\kappa_{\mathrm{Cu}}a_{\mathrm{Cu}}}{\varphi(c_1+c_0a_{\mathrm{Fe}}+c_2a_{\mathrm{Cu}})}\approx\begin{cases}\dfrac{\kappa_{\mathrm{Cu}}a_{\mathrm{Cu}}}{\varphi(c_1+c_2a_{\mathrm{Cu}})} & \text{if}\quad c_1+c_2a_{\mathrm{Cu}}\gg c_0a_{\mathrm{Fe}}\\ \dfrac{\kappa_{\mathrm{Cu}}a_{\mathrm{Cu}}}{\varphi(c_0a_{\mathrm{Fe}}+c_2a_{\mathrm{Cu}})} & \text{if}\quad c_0a_{\mathrm{Fe}}+c_2a_{\mathrm{Cu}}\gg c_1\end{cases}, \tag{S10}$$

which can be linearized as:

$$\frac{1}{s_{\mathrm{Cu}}}\approx\begin{cases}\varphi c_1\cdot\dfrac{1}{a_{\mathrm{Cu}}}+\varphi c_2 & \text{if}\quad c_1+c_2a_{\mathrm{Cu}}\gg c_0a_{\mathrm{Fe}}\\ \varphi c_0\cdot\dfrac{a_{\mathrm{Fe}}}{a_{\mathrm{Cu}}}+\varphi c_2 & \text{if}\quad c_0a_{\mathrm{Fe}}+c_2a_{\mathrm{Cu}}\gg c_1\end{cases} \tag{S11}$$

with

$$\varphi \equiv \frac{\kappa_{\mathrm{Cu}}}{\kappa_{\mathrm{Fe}}} \cdot \frac{A_{\mathrm{Fe}}^{m} K'_{m,\mathrm{Fe}}}{A_{\mathrm{Cu}}^{m} K'_{m,\mathrm{Cu}}}. \tag{S12}$$

Note that for the specific case of $A_{\mathrm{Fe}}^{m} = A_{\mathrm{Cu}}^{m}$, Eq. S5 leads to a linear scaling law, from which φ can be extracted:

$$\frac{a_{\mathrm{Fe}}^{m}}{a_{\mathrm{Cu}}^{m}} = \frac{K'_{m,\mathrm{Fe}} a_{\mathrm{Fe}}}{K'_{m,\mathrm{Cu}} a_{\mathrm{Cu}}} \quad \Rightarrow \quad \frac{s_{\mathrm{Fe}}}{s_{\mathrm{Cu}}} = \varphi \frac{a_{\mathrm{Fe}}}{a_{\mathrm{Cu}}}. \tag{S13}$$

Statistical Analysis

Correlation and regression analyses were performed with SPSS Statistics (R24.0.0.2; IBM, Armonk, NY, USA).

Supporting Data

In this study, we used the following XANES spectra published in the Materials Data Repository (MDR) XAFS Database:[78,79]

- XAFS spectrum of iron(II) oxide. MDR XAFS DB, National Institute for Materials Science (NIMS), Japan. DOI: 10.48505/nims.2045
- XAFS spectrum of iron disulfide. MDR XAFS DB, National Institute for Materials Science (NIMS), Japan. DOI: 10.48505/nims.2072
- XAFS spectrum of iron(II) sulfide. MDR XAFS DB, National Institute for Materials Science (NIMS), Japan. DOI: 10.48505/nims.2053
- XAFS spectrum of iron nitride. MDR XAFS DB, National Institute for Materials Science (NIMS), Japan. DOI: 10.48505/nims.2085
- XAFS spectrum of copper(I) sulfide. MDR XAFS DB, National Institute for Materials Science (NIMS), Japan. DOI: 10.48505/nims.2021
- XAFS spectrum of copper nitrate, hydrous. MDR XAFS DB, National Institute for Materials Science (NIMS), Japan. DOI: 10.48505/nims.1784
- XAFS spectrum of copper nitride. MDR XAFS DB, National Institute for Materials Science (NIMS), Japan. DOI: 10.48505/nims.1747

Supporting Tables

Table S1. βLG fragments of both conjugates that were not identified after digestion with combined trypsin and pepsin. Results were identical for PheoβLG and EuβLG. *A* and *B* indicate two protein isoforms. The primary binding site of dopamine quinones was His146 (indicated by **red** color).

Unidentified βLG fragments	
60–69	KWENDECAQK *(A)*
60–69	KWENGECAQK *(B)*
104–114	LFCMENSAAEPE
143–154	LPMHIRLSFNPT

Table S2. X-band EPR acquisition parameters.

Parameter	Value
Resonance frequency:	9.4193 GHz
Microwave power:	0.2 mW
Sweep range:	20–620 mT
Sweep time:	229.4 s
Modulation frequency:	100 kHz
Modulation amplitude:	0.5 mT
Temperature range:	10 K, 60 K, room temperature

Table S3. Results of LCA fits of Cu K-edge XANES spectra of PheoβLG-Cu-5% and PheoβLG-CuFe-1/10. The fits contained either 100% CuO or CuO plus a second standard with both components adding up to 100% (see Figure S5). The table lists the fraction of the second standard and the *R*-factor, a measure for the fit quality. It decreases with increasing agreement between fit and data. The uncertainty of the fractions of CuO and second standard amounted to approximately ±3% for all cases.

Second standard	Fraction (%)		*R*-factor (10^{-3})	
	PheoβLG-Cu-5%	PheoβLG-CuFe-1/10	PheoβLG-Cu-5%	PheoβLG-CuFe-1/10
—	—	—	5.4	7.0
Cu_2O	8	7	4.7	6.5
CuS	10	6	4.2	6.5
Cu_2S	7	4	4.4	6.6
$Cu(NO_3)_2{\cdot}3H_2O$	7	**22**	5.2	**4.8**
Cu_3N	12	8	4.2	6.3

Table S4. Results of proton relaxation and susceptibility measurements at room temperature. Longitudinal relaxation times were obtained from monoexponential fits to inversion-recovery turbo spin-echo (IR-TSE) data for pheomelanins and to the saturation-recovery data for eumelanins. Additional 3T IR-TSE results in the eumelanins agreed within the experimental accuracy.

**No.**	**Sample**	T_1 **[ms]**		T_2 **[ms]**				$\Delta\chi$ **[ppm]**
		3 T	7 T	T_2^{mono}	T_2^a	T_2^b	f_a	
PM	Matrix	1,922±19	—	746± 9				—
PB	βLG	1,870±15	—	521± 5				—
P0	PheoβLG	1,987±15	—	759± 7				–0.187±0.028
P1	PheoβLG-Cu-5%	1,547±16	—	435± 5				–0.186±0.023
P2	PheoβLG-Fe-10%	1,300±15	—	332± 4				–0.166±0.034
P3	PheoβLG-CuFe-1/1	1,308±12	—	422± 5				–0.168±0.029
P4	PheoβLG-CuFe-3/1	1,425±16	—	391± 4				–0.146±0.020
P5	PheoβLG-CuFe-5/1	1,318±11	—	423± 5				–0.172±0.026
P6	PheoβLG-CuFe-10/1	1,395±14	—	436± 5				–0.194±0.028
P7	PheoβLG-CuFe-1/3	1,307±12	—	402± 4				–0.141±0.020
P8	PheoβLG-CuFe-1/5	1,138± 9	—	359± 4				–0.149±0.035
P9	PheoβLG-CuFe-1/10	1,503±15	—	420± 5				–0.176±0.028
EM	Matrix	1,766±54	1,829± 5	578± 8	520±13	—[a]	0.86±0.08	—
EB	βLG	1,672±23	1,779± 4	555± 3	555± 1	—	~1	–0.155±0.028
E0	EuβLG	1,514±20	1,684± 9	585± 8	585± 1	—	~1	–0.218±0.036
E1	EuβLG-Cu-5%	1,479±17	1,708± 3	545±29	545± 1	—	~1	–0.137±0.029
E2	EuβLG-Cu-10%	1,397±17	1,554±17	464± 1	529±13	334±17	0.67±0.10	–0.137±0.030
E3	EuβLG-Fe-5%	1,304±16	1,535± 3	496± 3	496± 2	—	~1	–0.166±0.030
E4	EuβLG-Fe-10%	1,246±15	1,451±21	413±10	449± 4	252± 9	0.81±0.03	–0.176±0.031
E5	EuβLG-CuFe-1/1	1,022±11	1,308± 3	419± 6	465± 5	278±10	0.76±0.04	–0.154±0.023
E6	EuβLG-CuFe-3/1	1,094±11	1,349± 3	453±24	439± 9	—[a]	0.97±0.04	–0.046±0.097
E7	EuβLG-CuFe-5/1	1,289±16	1,506±17	438± 5	486±10	306±19	0.73±0.09	–0.167±0.027
E8	EuβLG-CuFe-10/1	1,250±14	1,413±22	455±14	449±17	—[a]	0.98±0.02	–0.157±0.030
E9	EuβLG-CuFe-1/3	910±11	1,178± 2	415±12	407± 9	—[a]	0.98±0.06	–0.149±0.051
E10	EuβLG-CuFe-1/5	987±11	1,247± 3	432±11	477± 4	273± 8	0.78±0.03	–0.152±0.028
E11	EuβLG-CuFe-1/10	832± 8	1,052± 7	318±13	377±13	257±11	0.51±0.19	–0.162±0.025

[a] In cases with $1 > f_a > 0.85$, the biexponential fit did not provide an estimate for T_2^b, but tended to minimize the residuals by fitting another contribution with $T_2 > T_2^a$.

Supporting Figures

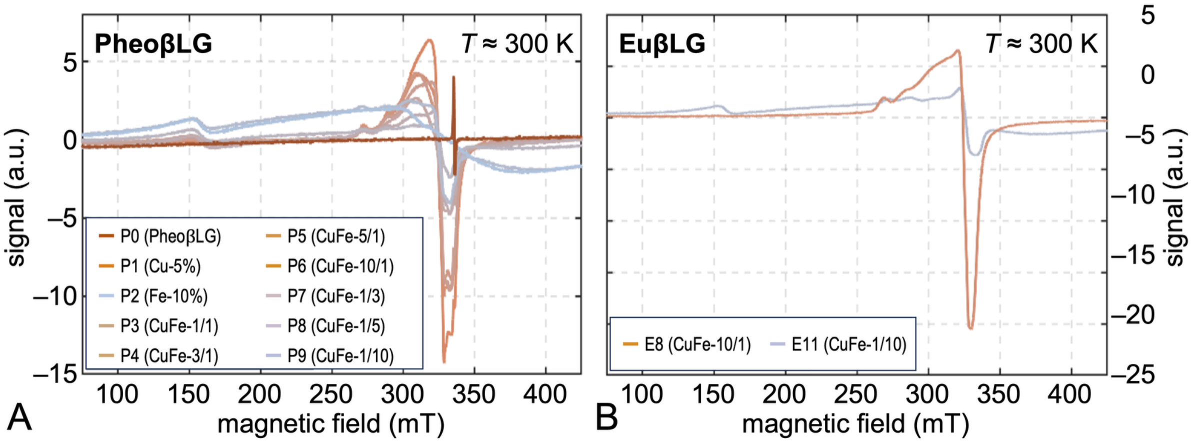

Figure S1. X-band EPR spectra of melanin-protein conjugates recorded at ~300 K. The variation of the metal content is indicated by the linfe color. The spectra have been scaled according to the receiver gain and the sample mass to ensure comparability of the recordings. (A) Spectra of PheoβLG-CuFe powders. (B) Spectra of EuβLG-CuFe powders.

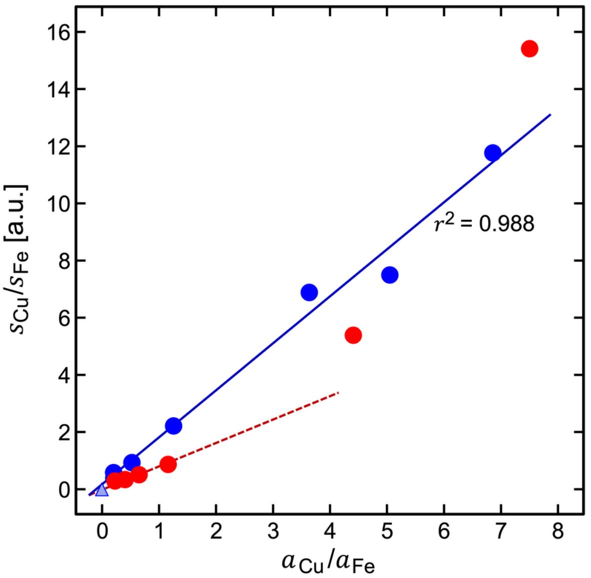

Figure S2. Variation of the EPR-active Cu/Fe ratio (measured at 10 K) with the total Cu/Fe ratio. The definition of the symbols is as in Figure 1. The data for PheoβLG-CuFe showed linear behavior (solid blue line) over the entire range. The data for EuβLG-CuFe increasingly deviated from a linear relation (dashed red line) at higher values of a_{Cu}/a_{Fe}.

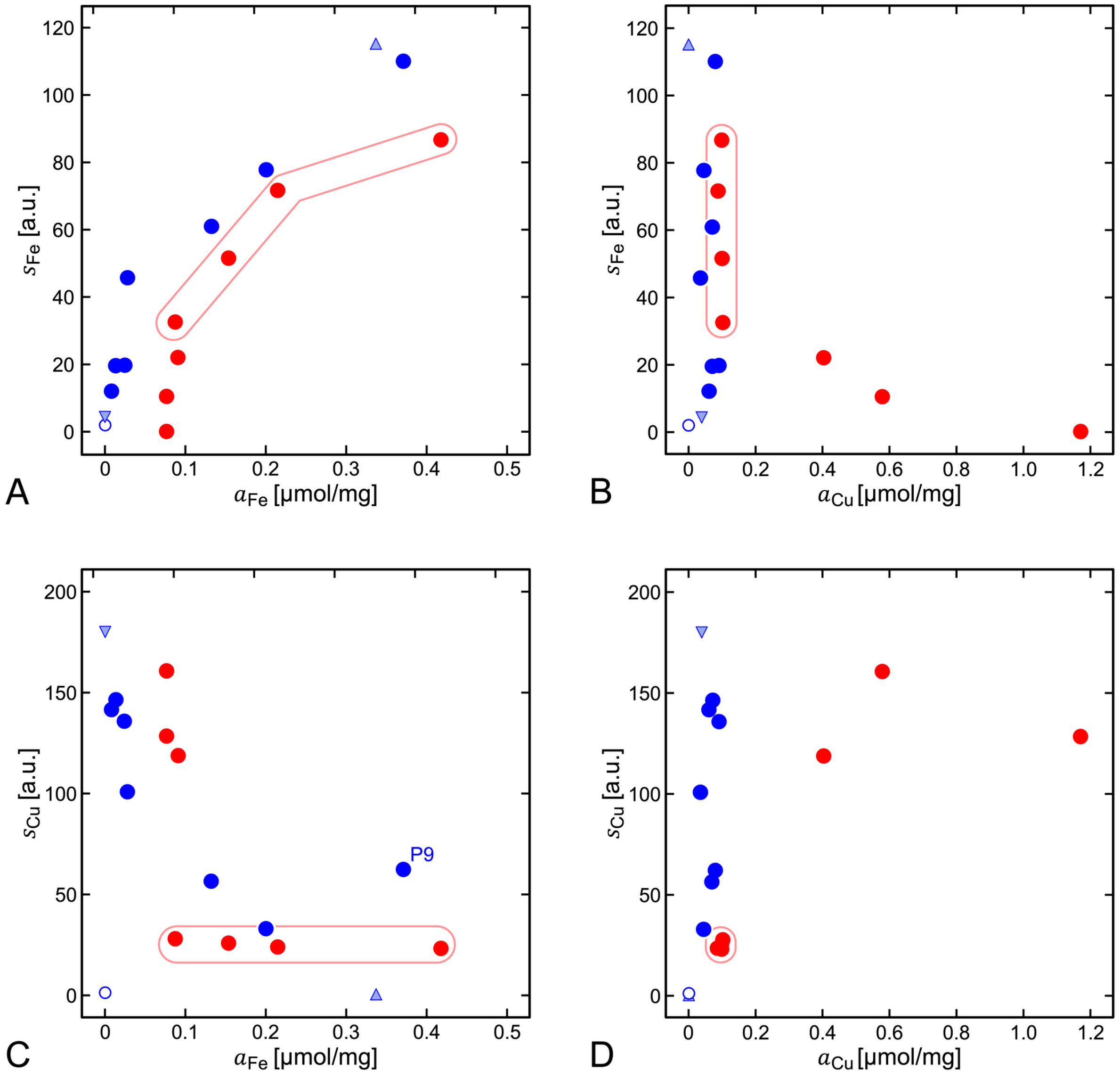

Figure S3. Dependencies of the EPR signals of Fe(III) and Cu(II) measured at 10 K on total metal contents. Blue and red symbols indicate data from PheoβLG-CuFe and EuβLG-CuFe, respectively, as in Figure 1. EuβLG-CuFe samples with large a_{Fe} variation at low a_{Cu} are enclosed by a solid line (compare Figure 1) (A) Variation of s_{Fe} as a function of a_{Fe}. (B) Variation of s_{Fe} as a function of a_{Cu}. (C) Variation of s_{Cu} as a function of a_{Fe}. (D) Variation of s_{Cu} as a function of a_{Cu}.

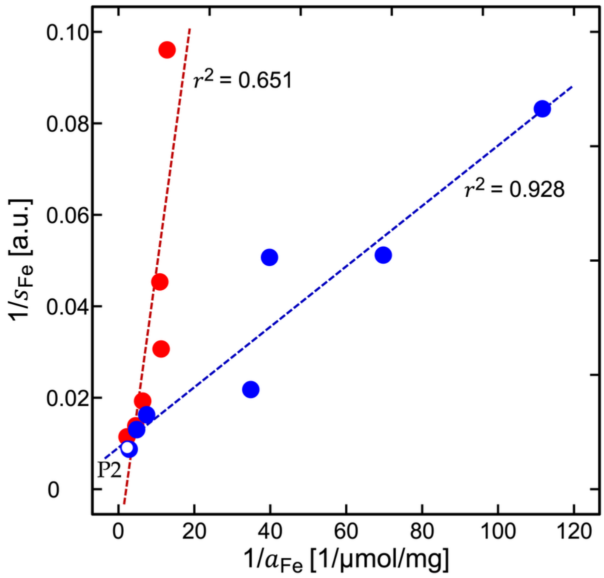

Figure S4. Lineweaver-Burk plot of the variation of $1/s_{\mathrm{Fe}}$ (measured at 10 K) with $1/a_{\mathrm{Fe}}$ without competition for binding sites. Blue and red symbols indicate data from PheoβLG-CuFe and EuβLG-CuFe, respectively, as in Figure 1. Results from fits to Eq. S9 for the limiting case of $c_1 + c_0 a_{\mathrm{Fe}} \gg c_2 a_{\mathrm{Cu}}$ are shown as dashed lines. This model is based on the assumption of negligible competition and yields a reasonable fit for PheoβLG-CuFe, whereas a systematic deviation is evident for EuβLG-CuFe. Improved fits were obtained in both PheoβLG-CuFe and EuβLG-CuFe upon consideration of competition effects as shown in Figure 5A.

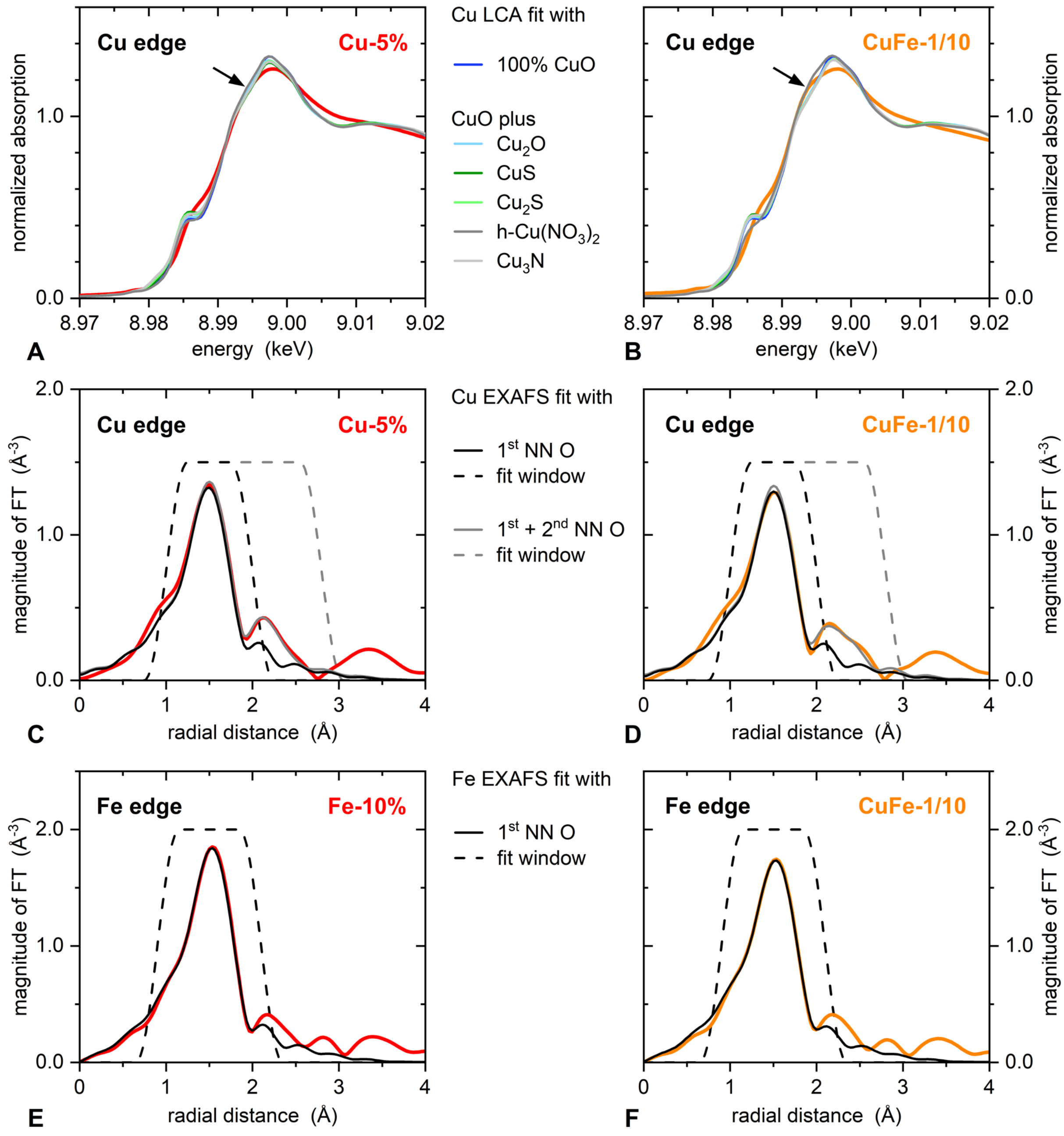

Figure S5. Quantitative analysis of the XAS data. LCA fits of the XANES spectra of (A) PheoβLG-Cu-5% and of (B) PheoβLG-CuFe-1/10 measured at the Cu K-edge. The fits contain either 100% CuO or CuO plus a second standard with both components adding up to 100% as summarized in Table S3. Also shown are fits of the Fourier-transformed EXAFS for (C) PheoβLG-Cu-5% and (D) PheoβLG-CuFe-1/10 measured at the Cu K-edge and for (E) PheoβLG-Fe-10% and (F) PheoβLG-CuFe-1/10 measured at the Fe K-edge. The path-fitting approach included scattering contributions from only first nearest neighbor (NN) oxygen atoms (black solid lines in C–F) or from first and second NN oxygen atoms (gray solid lines in C and D). Fitting ranges are indicated by dashed lines.

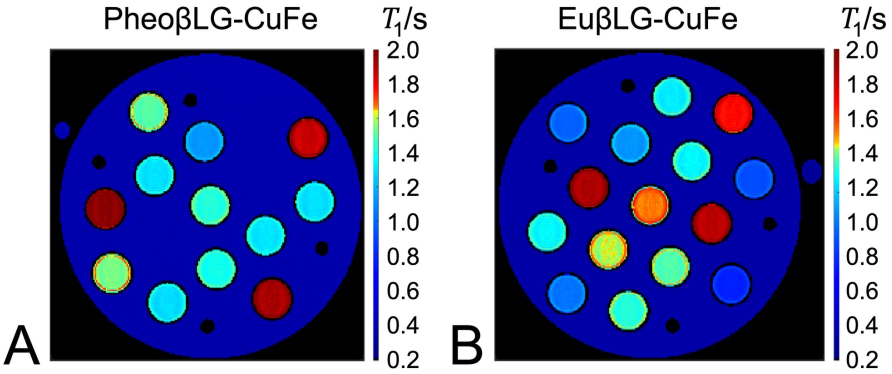

Figure S6. Water proton T_1 maps measured at 3 T and room temperature. (A) PheoβLG samples. (B) EuβLG samples. Small standard deviations (≤2%) within a circular region of interest (ROI) of ~80 voxels at each sample position indicated good homogeneity of the compositions. The space surrounding the samples was filled with agarose gel doped with 0.1% gadopentetate dimeglumine to achieve accelerated relaxation.

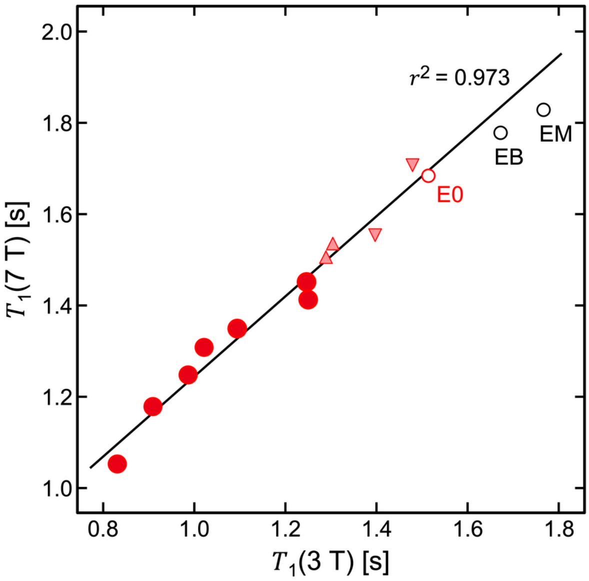

Figure S7. Comparison of water proton T_1 in EuβLG samples at 3 T and 7 T (both at room temperature). A strong linear correlation (r^2 = 0.973) was obtained for the entire range of compositions. The abbreviations and the symbols are defined in Table 1 and Figure 1, respectively.

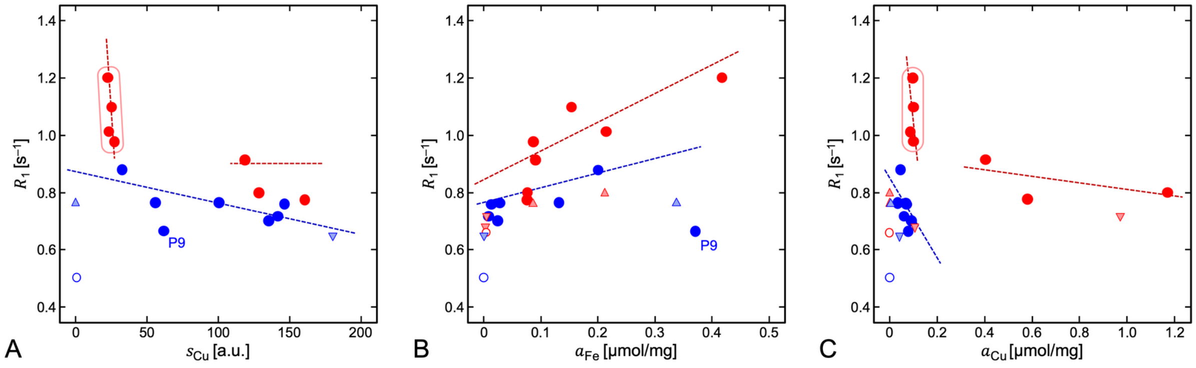

Figure S8. Proton longitudinal relaxation rates at 3 T and room temperature in dependence of the metal content. Blue and red symbols indicate pheomelanin and eumelanin conjugates, respectively, as in Figure 1. R_1 is plotted as a function of (A) the amount of EPR-active Cu(II) s_{Cu} (measured at 10 K) as well as **(B)** the total Fe content a_{Fe} and **(C)** the total Cu content a_{Cu}. As a guide to the eye, dashed blue and red lines show the change of R_1 explained by co-variation of the independent variable (i.e., s_{Cu}, a_{Fe} or a_{Cu}) and s_{Fe}. The expected R_1 variation due to the individual amount of EPR-active Fe(III) (see Figure 7A) yields good approximations to the data. EuβLG-CuFe samples with low a_{Cu} are enclosed by a solid red line.

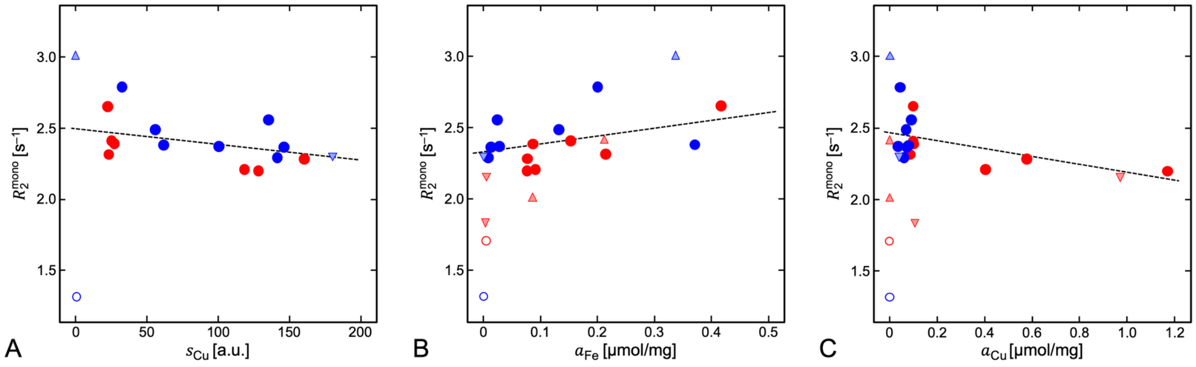

Figure S9. Proton transverse relaxation rates at 3 T and room temperature in dependence of the metal content. Blue and red symbols indicate pheomelanin and eumelanin conjugates, respectively, as in Figure 1. R_2^{mono} obtained by monoexponential fitting is plotted as a function of (A) the amount of EPR-active Cu(II) s_{Cu} (measured at 10 K) as well as (B) the total Fe content a_{Fe} and (C) the total Cu content a_{Cu}. As a guide to the eye, dashed lines show the change of R_2 explained by co-variation of the independent variable (i.e., s_{Cu}, a_{Fe} or a_{Cu}) and s_{Fe}. The expected R_2^{mono} variation due to the individual amount of EPR-active Fe(III) (see Figure 7B) yields good approximations to the data.
